# A Composite Based on *L*-Polylactide with Cu or CuO Nanoparticles: Physical Properties and Biological Activity

**DOI:** 10.3390/polym18080976

**Published:** 2026-04-17

**Authors:** Dmitriy A. Serov, Fatikh M. Yanbaev, Dmitriy E. Burmistrov, Ilya V. Baimler, Sergei O. Liubimovskii, Liudmila Y. Kozlova, Ivan A. Popov, Ann V. Gritsaeva, Polina A. Fomina, Lev R. Sizov, Valery A. Kozlov, Evgeny V. Kuzmin, Alexander V. Simakin, Maxim E. Astashev, Sergey V. Gudkov

**Affiliations:** 1 Prokhorov General Physics Institute of the Russian Academy of Sciences, Vavilov Str. 38, 119991 Moscow, Russia; dmitriy_serov_91@mail.ru (D.A.S.); dmitriiburmistroff@gmail.com (D.E.B.); ilyabaymler@yandex.ru (I.V.B.); nektoketon@gmail.com (S.O.L.); lus.kozlowa2011@kapella.gpi.ru (L.Y.K.); popovia2003@yandex.ru (I.A.P.); anngritsaeva@mail.ru (A.V.G.); polja.fomina@gmail.com (P.A.F.); leo.sizoff@yandex.ru (L.R.S.); avsimakin@gmail.com (A.V.S.); astashev@yandex.ru (M.E.A.); s_makariy@rambler.ru (S.V.G.); 2 Federal Research Center Kazan Scientific Center of the Russian Academy of Sciences, ul. Lobachevskogo 2/31, Tatarstan, 420088 Kazan, Russia; f.yanbayev@knc.ru; 3 Department of Fundamental Sciences, Bauman Moscow State Technical University, 5 2nd Baumanskaya St., 105005 Moscow, Russia; 4 Lebedev Physical Institute, Russian Academy of Sciences, 58 Leninskiy Av., 119991 Moscow, Russia; e.kuzmin@lebedev.ru

**Keywords:** nanoparticles, copper oxide, copper, polylactic acid, PLA, nanocomposite, biocompatibility, antibacterial, cytotoxicity, ROS

## Abstract

The development of biodegradable, biocompatible materials with inherent antibacterial properties, suitable for 3D printing, is a key challenge in modern materials science. Composites based on PLA and copper nanoparticles (NPs) are promising candidates for such a material. A protocol of the low-temperature incorporation of 0.1% Cu NPs or 0.1% CuO NPs into a PLA was developed. The dependence of the materials’ physicochemical properties on nanoparticle composition was evaluated. Cu and CuO NPs were synthesized via liquid-phase laser ablation and had sizes of 25 and 80 nm, with modal zeta potential values of +31 and +42 mV, respectively. The incorporation of Cu NPs enhances the tensile strength and Young’s modulus of PLA, and improves antibacterial properties. The PLA + 0.1% CuO or PLA + 0.1% Cu nanoparticles inhibited the growth of *E. coli* by ~60% and >80%, respectively. PLA + 0.1% Cu NPs destructed of bacterial cell walls. The antibacterial action mechanisms are an 8-oxoguanine and LRPS generations. The obtained materials did not exhibit cytotoxic effects against normal human fibroblasts, did not alter the pH or redox potential of water, and did not release of Cu^2+^ in concentrations toxic to humans. The material PLA + 0.1% Cu NPs is the most optimal. This material may find applications in food production and biomedical applications.

## 1. Introduction

Polymer materials are widely used in virtually every area of human economic activity and everyday life: mechanical engineering, aviation, construction, electronics, cosmetics, medicine and biomedicine. The wide range of applications for polymer materials is due to the diversity of their carbon chain structures [1]. The global market for polymer products is worth over 1.3 × 10^12^ USD [2].

The widespread use of polymer coatings in both industry and everyday life has led to a serious environmental pollution problem. Global annual plastic production exceeds 400 million tons, and since the mid-20th century, a total of over 9 billion tons of plastic have been produced. The total volume of plastic waste exceeds 8 million tons and accounts for over 85% of all marine litter [3,4].

One of the reasons why plastics pose a threat to the environment and why plastic waste accumulates so rapidly is their long decomposition time in the natural environment. This varies depending on the polymer, ranging from 2 to 400 years for polylactone and polystyrene, respectively [5,6]. Over 50% of this plastic packaging is used only once before being disposed of and is not recycled [7]. In addition, many traditional polymers are used in the manufacture of multilayer, laminated and composite packaging, among which aluminum foil or silicon dioxide is incorporated into the middle layer to improve barrier properties. At the end of their service life, only a small proportion of this waste is recycled, as the process of separating the multiple layers is costly, and the majority of plastic waste, particularly from the food packaging sector, is disposed of in landfill. Furthermore, food products often contaminate plastic containers, making their reuse or recycling even less economically viable and leading to large volumes of end-of-life waste without recycling [8,9,10]. Unfortunately, recycling technologies do not yet allow for a sufficient rate of plastic waste recycling. Only up to 14% of the plastic products manufactured worldwide are recycled [11]

One way to tackle the problem of plastic waste accumulation is to use biodegradable plastics [12,13]. One of the most promising candidates for a biodegradable plastic with a wide range of applications is polylactic acid (PLA). It offers a number of advantages that make it an attractive option: biodegradability and safety in relation to the environment and medical applications. PLA breaks down into CO_2_ and water. Furthermore, PLA is non-toxic and biocompatible [14], and offers excellent performance characteristics, enabling it to be moulded into a variety of shapes and ensuring a long service life. PLA has the potential to be used for creating three-dimensional scaffolds in surgery, for example in the restoration of alveolar bone fragments and other procedures [15,16,17].

The purpose of food packaging is not only to store food products, but also to protect them throughout the supply chain (from the point of production to the table) from external abiotic and biotic agents by preventing chemical or microbiological contamination, which could lead to deterioration in consumer properties and/or a reduction in shelf life [18]. This has led to a need within the food industry to develop new packaging systems to ensure both the safety and quality of packaged products [19].

The main requirements for packaging vary depending on the application and intended use. The most common properties include barrier protection against gases (CO_2_, N_2_, O_2_, or volatile organic compounds) and moisture, adequate mechanical properties (flexibility or rigidity), airtightness, heat resistance, transparency or opacity depending on the application, protection against condensation, suitability for 3D printing, resistance to light, acids and fats, and, last but not least, cost [20].

One solution for the production of single-use and short-lived packaging that is also environmentally friendly is biodegradable and compostable bioplastics, which can play a crucial role and offer great potential for the production of everything from flexible films to rigid plastics; these materials combine renewable origins with the necessary condition of biodegradability at the end of their service life due to their susceptibility to microbial action in nature [10,21,22]. Biopolymers are most widely used in food and non-food packaging: polybutylene succinate-co-adipate (PBSA), polybutylene adipate terephthalate (PBAT), polyhydroxyalkanoates (PHAs), and PLA. In addition to biomedicine, PLA can be used in the production of packaging materials for the food and agricultural industries [23]. Unfortunately, biodegradable polymer materials have a critical drawback: they are highly susceptible to bacterial contamination during use. This is a critical issue for both medical and food applications [24]. The incorporation of NCPs into a polymer matrix, including PLA, is one way of imparting antibacterial properties to medical devices and packaging materials in the food industry [25,26].

Copper (Cu) and copper oxide (CuO) nanoparticles (NPs) are widely used in the fields of chemistry, catalysis, medicine, wastewater treatment and agriculture [27,28]. Copper Cu and CuO possess pronounced antibacterial properties [29,30]. The main mechanisms of the antibacterial action of Cu NPs are direct physical damage to cell walls, genotoxic effects and the inhibition of bacterial enzyme activity through the release of Cu^+^ or Cu^2+^ ions, the disruption of bacterial metabolism, the generation of reactive oxygen species (ROS) and the induction of oxidative stress, which enhances all the mechanisms described above [31,32,33,34,35]. In particular, PLA + Cu NP-based composites could be used in the production of packaging materials for meat products [36]. Composites based on PLA + Cu NPs and plant essential oils have demonstrated excellent performance characteristics and pronounced antibacterial properties [37]. Antibacterial composites based on PLA + Cu NPs can also be used for 3D printing [38]. The addition of copper NPs with different compositions makes it possible to modify and fine-tune the mechanical and antimicrobial properties of polymers [39].

Despite significant advances in the creation of antibacterial composites based on PLA and copper-containing NPs, unresolved issues remain. The main challenges in creating antibacterial materials based on PLA + Cu or CuO NPs, based on research data for 2023–2025, are listed below (Table 1).

The production of Cu and CuO nanoparticles by laser ablation is a rapid and cost-effective method that enables the production of nanoparticles with a narrow size distribution [48]. Furthermore, the production of low-frequency components using laser ablation does not require the use of chemical reagents or the removal of reaction products, which is characteristic of conventional chemical synthesis methods [29]. In addition to their high performance, barrier and antibacterial properties, the new composite materials must be non-toxic to humans and animals.

In this study, we prepared composites based on PLA, Cu PNs and CuO NPs. The nanoparticles were synthesized using the liquid-phase laser ablation method, and the incorporation of nanoparticles into the polymer matrix was carried out using PLA via a low-temperature method developed by us. For the resulting polymer composites, we evaluated the dependence of physicochemical properties, mechanical properties, degree of crystallinity, antibacterial activity and cytotoxicity against human cells on the type of incorporated Cu or CuO nanoparticles. In addition, we investigated the potential mechanisms of the antibacterial activity of the composites: the release of copper ions, pH change, ROS generation, and oxidative modification of biopolymers (DNA and proteins)

## 2. Materials and Methods

### 2.1. Cu or CuO NP Synthesis

The synthesis of nanoparticle colloids was carried out using methods involving laser ablation of a solid Cu (99.99%) target in liquid, followed by laser fragmentation of the nanoparticle colloids. In the particle synthesis experiments, a conventional liquid-phase laser ablation setup was employed. A solid target of the selected material was fixed to the bottom of a 50 mL glass cuvette and filled with the working fluid so that the thickness of the fluid layer between the target surface and the fluid was 2–3 mm. To obtain CuO NPs, deionized water (0.05 μS/cm) was used as the working fluid. For the synthesis of Cu NPs, 99% acetone (Lenreaktiv, Moscow, Russia) was used as the working fluid. A pulsed Nd:YAG laser NL300 (Ekspla, Vilnius, Lithuania) with the following parameters was used for the synthesis of CuO and Cu nanoparticles: λ = 1064 nm, f = 1 kHz, τ = 4 ns, and Ep = 2 mJ. The laser spot size at the focal point was 200 μm. The galvanometer scanner LScanH (Ateco-TM, Moscow, Russia) and an F-Theta lens with a focal length of 90 mm were used. The focused beam was moved along the surface of the target. The trajectory of the laser beam consisted of several parallel lines inscribed within a 1 × 1 cm square. The line density was 70 lines per millimetre. The beam movement speed using the scanning system was 3000 mm/s. The typical duration of laser ablation was 30 min [49].

### 2.2. Assessment of the Physicochemical Properties of Cu or CuO NPs

A Libra 200 FE HR transmission electron microscope (TEM) equipped with EDX module (Carl Zeiss, Jena, Germany) was used to obtain images of the NPs, study their morphology and assay element composition. Gold microgrids (Labtech, Moscow, Russia) were used to prepare the nanoparticles for TEM microscopy. The distributions of nanoparticles by hydrodynamic diameter and zeta potential were determined using a Malvern Zetasizer Ultra particle analyzer (Malvern Panalytical Ltd., Malvern, Worcestershire, UK) with 10 × 10 mm transparent quartz cuvettes fitted with a ZEN1002 electrode (Malvern Panalytical Ltd., Malvern, UK). Data analysis was performed using ZS Xplorer v. 3.2 software (Malvern Panalytical Ltd., Malvern, Worcestershire, Malvern, UK). Absorption spectra of the NPs colloids in the visible and UV ranges were recorded using a CINTRA 4040 dual-beam spectrophotometer (GBC Scientific Equipment Pty Ltd., Keysborough, VIC, Australia). The optical density was measured in the range of 200–800 nm with a step of 1 nm. The spectra were measured in quartz cuvettes with a volume of 3 mL. The optical path length was 1 cm. The absorption spectra of the working liquid used in laser ablation were used as reference spectra.

### 2.3. Doping of PLA with Cu and CuO

The incorporation of NPs into a PLA polymer matrix was carried out using the low-temperature method described below. Firstly, 0.1% Cu nanoparticles were synthesized in acetone and added directly to the dissolved PLA (see below) without further modification. The final concentrations of Cu NPs were 0.001, 0.01, or 0.1% (*w*/*w*). CuO NPs obtained by laser ablation in water were transferred to 99.9% dichloromethane (DCM) prior to incarnation into PLA (Komponent-Reaktiv, Moscow, Russia). Twenty millilitres of the CuO NP suspension in deionized water were transferred to a centrifuge tube and centrifuged at 7000× *g* and 22 °C for 40 min. After centrifugation, the water was removed and replaced with 20 mL of 99.9% isopropanol (Lenreaktiv, Moscow, Russia). The CuO nanoparticles were mixed in isopropanol using a Vortex V-1 plus (Biosan, Riga, Latvia), followed by ultrasonic treatment for 3–5 min at 40 kHz in an ultrasonic bath PS-20A (Digital Pro, Beijing, China). Centrifugation was repeated, and isopropanol was replaced by dichloromethane (DCM). After the second centrifugation, as much solvent as possible was removed so that 200 µL of the CuO NP suspension in dimethyl sulfoxide (DMSO) remained at the bottom of the tube. PLA 99.9% (PRO SynTech, Moscow, Russia) filaments weighing 25× *g* were cut with scissors and placed in 60 mL of DCM; the container was sealed and stirred for 5 h at 65 °C and 350 rpm. Then, 0.1% Cu or 0.1% CuO was added to the PLA solution at a final concentration of 0.1% (*w*/*w*). The resulting mixture was stirred twice for 5 min at 350 rpm and subjected to ultrasonic treatment for 3–5 min at 40 kHz and 22 °C. The resulting mixture was poured into special moulds made from Creality Standard Resin PLUS LCD 1 photopolymer resin (Creality, Moscow, Russia) with the 3D printer Saturn (Elegoo, Shenzhen, China). A PLA solution in DCM without NPs was used as a control. The samples were dried in a fume cupboard for 24 h. To investigate physicochemical properties, the generation of ROS, 8-oxoguanine and long-lived reactive protein species (LRPS), the releasing of Cu^2+^, changes in pH and Rexod, antibacterial activity, and cytotoxicity, square samples measuring 10 × 10 × 1 mm were prepared. To assess mechanical properties, samples 1 cm wide, 1 mm thick and 80 mm long were prepared.

### 2.4. Assessment of the Physicochemical Properties of Polymeric Materials

Absorption spectra of the materials in the UV–visible range were obtained using a CINTRA 4040 dual-beam spectrometer (GBC Scientific Equipment Pty Ltd., Keysborough, VIC, Australia). The surface homogeneity of polymeric materials at the micro-scale was assessed using atomic force microscopy (AFM) without prior sample preparation, employing an NT-MDT microscope (LLC, Zelenograd, Russia). Fragments of samples with an area of 9 × 9 μm were analyzed. The distribution of Cu NPs (*n* ≈ 0.56 for λ = 500 nm) and CuO NPs (*n* ≈ 2.65 for λ = 500 nm) within the PLA polymer matrix (*n* ≈ 1.45 for λ = 500 nm) was assessed using modulation–interference microscopy with an MIM-321 system at a wavelength of 632 nm (Amphora Labs, Moscow, Russia). Tensile tests on the cast samples were performed using a WDW-5S universal testing machine (Hongtuo, Binzhou, China). The hydrophobicity and hydrophilicity of the materials were assessed based on the contact angle measured using the sessile drop method [50]. A 5 µm drop of deionized water was applied to material samples measuring 80 × 10 × 1 mm. High-contrast photographs of the drops were obtained using a setup based on an SDU3-C264 CMOS camera (SpetsTeleTechnika, Moscow, Russia), a movable stage with micromanipulators, a Wanptek DPS605U laboratory power supply, and an LED illuminator with a diffuser. Photographs of the droplets were taken at a temperature of 25 °C and a relative humidity of 50%. Initial image processing was carried out using CamView V2.3 (e-con Systems, Suite, Fremontm, CA, USA) software. Further image analysis was performed using ImageJ 1.54p software (Fiji) (National Institutes of Health University of Wisconsin, Bethesda, WI, USA) and the DropSnake plugin Version 2.1 (march 2006 by Aurelien Stalder). During the analysis, the left and right contact angles were determined for each droplet. For each experimental condition, 20 independent measurements were taken.

### 2.5. Assessment of the Degree of Crystallinity

The Raman spectra of all samples were recorded at room temperature using a Senterra II confocal Raman microscope (Bruker Optics, Billerica, MA, USA) at excitation wavelengths of 532 and 785 nm. The spectra were recorded at 180° scattering with a spectral resolution of 1.5 cm^−1^. At an excitation wavelength of 532 nm, the laser power was 25 mW, and at an excitation wavelength of 785 nm, it was 100 mW. The excitation and scattered radiation were focused using a 20× objective (numerical aperture 0.40) for films, colloids and liquids, and a 4× objective (numerical aperture 0.10) for colloids and liquids. The laser spot diameter on the sample surface was 10 µm at an excitation wavelength of 532 nm and 12 µm at an excitation wavelength of 785 nm in the case of the 20× objective.

The samples were placed on a metal plate during the recording of the Raman spectra of films. The metal dish was covered with a thin cover glass during the recording of the spectra of liquids and colloids. It was verified that the Raman spectrum of the cover glass did not exhibit any notable features in the region of the Raman lines used for the analysis of the samples under investigation. The spectrum recording time was selected individually for each sample to ensure a good signal-to-noise ratio.

Raman spectra were recorded at several (3–5) points on the surface of each film to ensure that the film structure was uniform. Analysis showed that all spectra measured at different points on the surface of a single sample were virtually identical. Consequently, the spectrum used for analysis was obtained by averaging the spectra recorded at different points on the surface.

To verify that the structure of the sample did not change during the spectral recording process due to heating by the laser beam, spectra were recorded several times at a single point on the surface of each sample. All of these spectra proved to be identical.

In all figures, the experimental Raman spectra are shown after subtraction of the fluorescence background, which was performed using non-linear functions. For all Raman spectra recorded at an excitation wavelength of 785 nm, the fluorescence background was negligible. However, when Raman spectra were recorded at an excitation wavelength of 532 nm, a more pronounced fluorescence background was observed. It is assumed that this background is due to the presence of impurities remaining after synthesis. The relative error in measuring the ratio of the peak intensities of the Raman lines in the spectra recorded at a wavelength of 785 nm was estimated to be 5–7%. The degree of crystallinity of the samples was assessed according to the *I*_411_/*I*_874_ ratio of the peak intensities of the Raman lines in the spectrum at frequencies of 411 and 874 cm^−1^.

### 2.6. Assessment of Cu^2+^ Release from the Materials Obtained and Changes in Water Properties Following Contact with the Materials

To assess the release of Cu^2+^ ions from PLA + Cu NPs or PLA + CuO NPs, the samples were placed in 10 mL of deionized water (with a specific electrical conductivity of 0.1 μS/cm, obtained using the ‘DV-5-OSMOS’ filtration system (Tsvet-khrom, Moscow, Russia) in plastic vials. Water without the addition of samples was used as a control. The water containing the samples and the control water were incubated for 72 h under constant stirring at 300 rpm and a temperature of 37 °C. Measurements of specific electrical conductivity, pH and redox potential were performed before and after incubation using the Seven Excellence multifunctional pH–redox–conductivity metre (Mettler Toledo, Küsnacht, Switzerland). The concentration of Cu^2+^ ions (*C*_Cu_^2+^) was estimated from the change in electrical conductivity using Formula (1):

(1) $$C_{{Cu}^{2+}}=\frac{\kappa }{{\frac{1}{2}\lambda }_{{Cu}^{2+}}}$$

where *κ* is the measured specific electrical conductivity of water at 25 °C, λ_Cu_^2+^ is the mobility of Cu^2+^ ions at 25 °C, and 1/2 is the factor for converting the equivalent concentration of Cu^2+^ to molar concentration.

### 2.7. Assessment of Formation of Reactive Species in Water and Aqueous Solutions

H_2_O_2_ was selected as the most long-lived ROS and the OH radical as the most reactive ROS. To assess the effect of PLA, PLA + Cu NP and PLA + CuO NP samples on H_2_O_2_ generation, the samples were incubated in 10 mL of deionized water for 3 h at 40 °C. After incubation, the H_2_O_2_ concentration was determined by using a chemiluminescent method based on the rate of the oxidation reaction of 50 μM p-iodophenol in the presence of 10 nM horseradish peroxidase in 1 mM Tris-HCl buffer at pH 8.5. Measurements were performed on a ‘Biotox-7A-USE’ chemiluminescence analyzer (Engineering Centre-Ecology, Moscow, Russia). H_2_O_2_ concentrations were calculated using pre-established calibration curves. The lower detection limit for H_2_O_2_ did not exceed 100 pM. The generation of OH radicals was assessed after incubating the samples in 10 mL of an aqueous CCA solution for 2 h at 80 °C. The concentration of OH radicals was assessed based on the intensity of the CCA oxidation product, 7-hydroxycoumarin-3-carboxylic acid, at wavelengths of 400/450 nm with the 8300 spectrofluorometer (JASCO, Hachioji, Tokyo, Japan). The concentrations of OH radicals were calculated using pre-established calibration curves. In all experiments, control measurements were carried out without samples to exclude background signals. Each analysis was performed in triplicate. The methods are described in more detail in previous works [51,52].

The rate of 8-hydroxyguanine formation was assessed following a 3 h incubation of the samples in an aqueous DNA solution at a concentration of 0.35 mg/mL. The concentrations of 8-oxoguanine in the solutions were determined by ELISA using specific primary monoclonal antibodies against 8-oxoguanine (1:2000). Non-specific binding was blocked with a 1% solution of skimmed milk in Tris-HCl buffer (pH 8.7) supplemented with 0.15 M NaCl for 1 h at room temperature. Incubation with primary antibodies was carried out for 3 h at 37 °C. After washing, secondary antibodies conjugated with horseradish peroxidase (1:1000) were added, followed by incubation for 90 min at 37 °C. 18.2 mM ABTS (2,2′-azino-bis-(3-ethylbenzothiazoline-6-sulfonic acid)) (Sigma, St. Louis, MO, USA) was used as the staining substrate in the presence of 2.6 mM H_2_O_2_ in 75 mM citrate buffer at pH 4.2. The reaction was stopped by the addition of 1.5 mM sodium azide [53]. Optical density was measured at 405 nm using a Feyond-A400 microplate reader (Ausheng, Hangzhou, China). To assess LRPS generation, 10 × 10 × 0.5 mm composite material plates were incubated for 120 min in 10 mL of 0.1% aqueous BSA colloid (Sigma, St. Louis, MO, USA) at 40 °C. The LRPS concentration was estimated based on the chemiluminescence intensity of the solution after incubation at 40 °C for 2 h and a 30 min incubation at room temperature. Measurements were performed using a Biotox-7A chemiluminometer (Engineering Centre ‘Ecology’, Moscow, Russia) in 20 mL polypropylene vials (Beckman, Brea, CA, USA) in the dark at 25 °C. BSA without heating was used as a control [54].

### 2.8. Assessment of Antibacterial Activity

A culture of the Gram-negative bacterium *Escherichia coli* (strain BL21) was used to assess antibacterial activity. The culture was grown in LB broth (Diam, Moscow, Russia) at 37 °C with constant stirring. Prior to the experiments, the OD_600_ of the culture suspension was determined to establish the concentration of *E. coli*. The test samples were added to the wells of 24-well plates (TPP, Trasadingen, Switzerland); no material was added to the control wells. Next, 1 mL of LB containing *E. coli* at a concentration of 10^6^ cells/mL was added. LB without bacteria was added to some of the wells. Growth curves were plotted using a Feyond-A400 plate reader (Allsheng, Hangzhou, China) over a 24 h period with continuous agitation at 200 rpm and a temperature of 37 °C to assess the dynamics of bacterial growth. To assess bactericidal activity, samples were extracted immediately after plotting the growth curves and stained with 4 µM propidium iodide (PI) (Lumiprobe, Hunt Valley, MD, USA) for 60 min in the dark. The stained suspensions were transferred to sterile centrifuge microtubes and analyzed using a Longcyte CLQC-281 flow cytometer (ChBio, Nantou, China). The concentration of bacterial cells in the suspension was assessed using a threshold method when constructing SS-FS histograms. Dead bacterial cells were identified by the increased intensity of PI fluorescence using the threshold method. For each experimental variant, at least 3 independent measurements were performed. A total of 50–100 thousand bacterial cells were analyzed in each measurement [55].

### 2.9. Assessment of Intracellular ROS Generation in Bacterial Cells

*E. coli* cultures were incubated as described in the previous section to assess intracellular ROS production. The bacterial cell concentration was adjusted to 10^6^ cells/mL after 24 h of incubation. The suspension (1 mL) was centrifuged for 10 min at 2000× *g* and resuspended in PBS. Cells were stained with 20 µM H_2_DCFDA (Lumiprobe, Hunt Valley, MD, USA) for 60 min at room temperature in darkness. After staining, cells were washed with PBS. The cell suspension volume was adjusted to 1 mL with fresh PBS and analyzed on a Longcyte CLQC-281 flow cytometer (ChBio, Nantou, Taiwan). The intensity of DCFDA fluorescence was measured at 488/520 (Ex/Em) wavelengths. The proportions of cells with DCFDA fluorescence intensity upper control values (cell with oxidative stress) were measured by using the threshold method. For each experimental variant, 6 independent measurements were performed. A total of 50–100 thousand bacterial cells were analyzed in each measurement.

### 2.10. Assessment of Cytostatic and Cytotoxic Effects

The cytotoxic activity was assessed using the human fibroblast cell line HSF (the cells were kindly provided by the Collection of Human Cell Cultures for Biomedical Purposes at VILAR). Cells from 10 to 12 passages were used in this study. Cell culture and subculturing were carried out according to standard protocols in DMEM/F12 medium supplemented with 10% FBS, 2 mM L-glutamine, 25 units/mL penicillin and 25 μg/mL streptomycin (PanEco, Moscow, Russia). Prior to the experiment, cells were detached from the substrate using a solution of trypsin and EDTA, and cell counts were performed in a Neubauer chamber. Pre-sterilized round cover glasses (2 h, 120 °C) were placed into the wells of a 6-well culture plate, and 200 μL of a suspension containing 200,000 cells in culture medium was applied to them. The plates were incubated in a CO_2_ incubator at 37 °C and 5% CO_2_. After incubation, the cells were double-stained with the vital dye 2′-(4-ethoxyphenyl)-5-(4-methyl-1-piperazinyl)-2,5′-bi-1H-benzimidazole trihydrochloride Hoechst 33342 (Lumiprobe, Hunt Valley, MD, USA) for 30 min at 37 °C. Following staining with 5 µg/mL Hoechst 33342, the cells were washed with sterile PBS and stained with 2 mM PI (Lumiprobe, Hunt Valley, MD, USA) immediately prior to analysis. Cell viability and morphology were assessed using fluorescence microscopy on a DMI4000 B microscope (Leica, Wetzlar, Germany), equipped with an SDU-285 digital camera (SpetsTeleTechnika, Moscow, Russia). WinFluorXE software v 3.8.7 8-12-16 (J. Dempster, Strathclyde Electrophysiology Software, University of Strathclyde, Glasgow, UK) was used for data acquisition. Analysis of viability, confluence, and cell and nuclear areas was performed using ImageJ2 (Fiji) software (National Institutes of Health University of Wisconsin, Bethesda, Waukesha, WI, USA). At least five samples were analyzed for each experimental condition. Between 250 and 500 cells were analyzed in each sample. The method is described in more detail in the paper [56].

### 2.11. Statistical Processing

The normality of sample distributions was tested using the Shapiro–Wilk test. The data obtained were presented as the mean values ± standard error (SE) or median with percentiles 25 and 75%. Statistical hypotheses were tested using the Mann–Whitney U test or two-sample two-tailed *t*-test. Differences were considered statistically significant at a significance level of *p* < 0.05. The exact sample sizes are indicated in the captions to the corresponding figures. Cohen’s d values were additionally calculated (Tables S1–S8).

## 3. Results and Discussion

### 3.1. Physicochemical Properties of Nanoparticles

**Figure 1:**
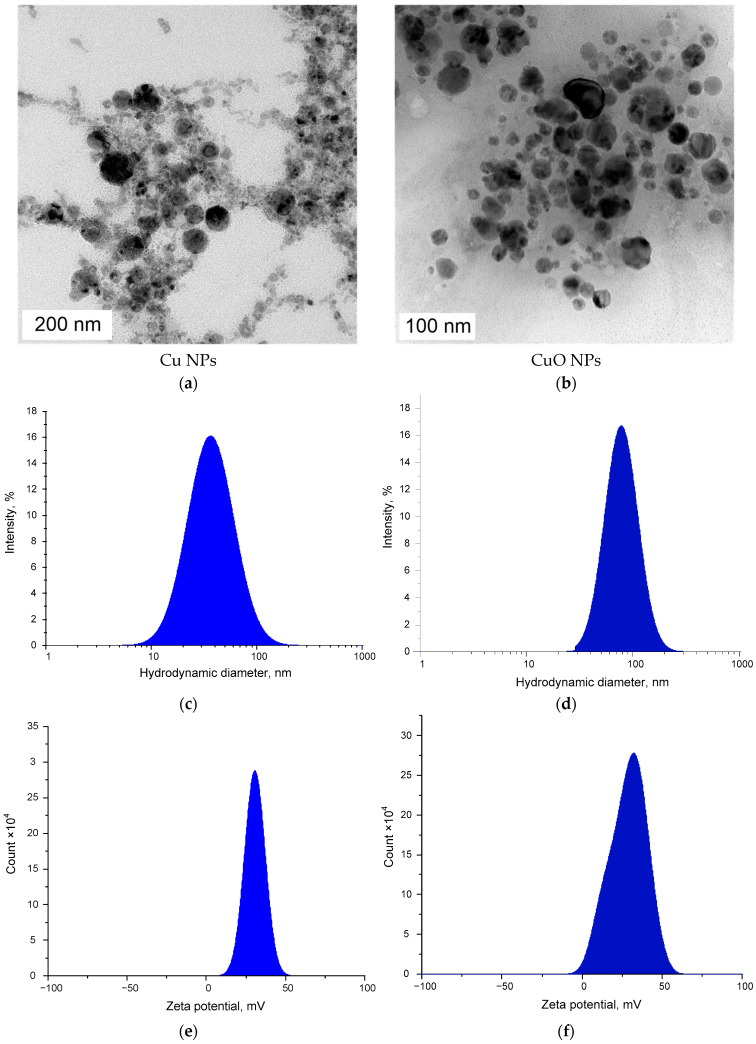
Physical properties of synthesized by laser ablation NPs: TEM images of Cu (**a**) or CuO NPs (**b**), distribution by hydrodynamic diameter Cu NPs (**c**) or CuO NPs (**d**), and distribution by zeta potential Cu NPs (**e**) or CuO NPs (**f**).

Cu NPs obtained by laser ablation in acetone exhibited a quasi-spherical morphology (Figure 1a) and a narrow monomodal distribution with respect to the hydrodynamic radius (Figure 1c). The size distribution of Cu nanoparticles by hydrodynamic radius exhibited a peak at 25 nm and a half-width of 10–80 nm. CuO nanoparticles produced by laser ablation in water also exhibited a quasi-spherical morphology (Figure 1b).

For nanoparticles obtained by laser ablation, the quantitative dependence of the modal values of the zeta potential and hydrodynamic diameter on the laser power density and/or the type of solvent used has been described [57,58,59]. In some cases, the half-width of the NP size distribution is narrow and can reach 10–15 nm or less [58]. In the case of chemical synthesis via the co-precipitation method, the variation in NP sizes can reach more than 100 nm, and the use of surfactants is required to ensure a narrow distribution of NPs by diameter and/or zeta potential [60].

**Figure 2:**
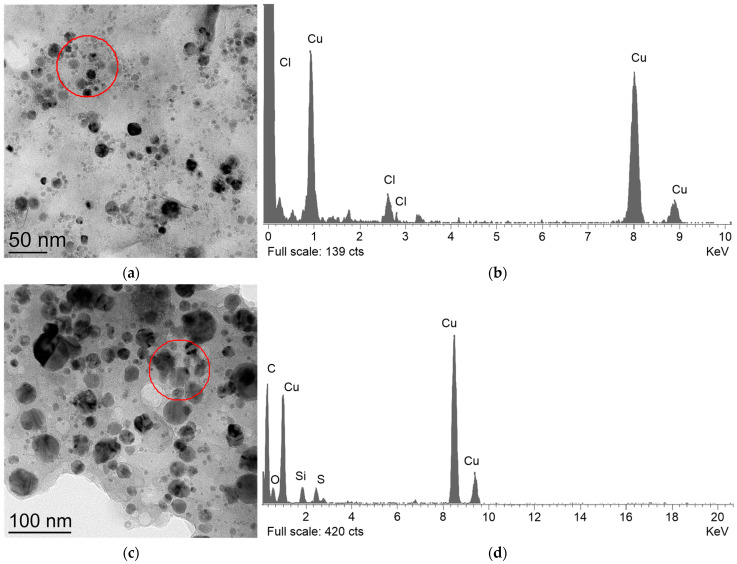
Elemental composition of NPs: TEM images of Cu (**a**) or CuO NPs (**c**) and EDX spectra of Cu (**b**) or CuO NPs (**d**). The highlighted areas (red circles) indicate the sites where the NP composition was analyzed using EDX spectroscopy.

The distribution of CuO NPs by hydrodynamic diameter was broader than that of Cu NPs. The hydrodynamic diameter of CuO NPs exhibited a monomodal distribution (Figure 1d) with a peak of 80 nm and a half-width of between 40 and 105 nm. Thus, the CuO nanoparticles had an average size approximately twice that of the Cu nanoparticles. The zeta potential values for both types of nanoparticles followed a monomodal distribution (Figure 1e,f). The peak values of the zeta potential for Cu and CuO NPs were +31 and +42 mV, respectively. CuO NPs exhibited a broader distribution of the zeta potential compared to Cu PNs: half-widths of 1–50 and 20–45 mV, respectively. Cu and CuO NPs were prepared via liquid-phase laser ablation. This method enables the preparation of metal and metal oxide nanoparticles with a narrow size distribution in the zeta potential [61,62]. The size distribution of Cu NPs in the range of 10–100 nm is consistent with data obtained by other researchers during the synthesis of nanoparticles using the laser ablation method [61]. The size of CuO NPs, which is less than 200 nm, is also consistent with data in the literature [63]. Consequently, the laser ablation method enables the production of copper and copper oxide NPs with narrow, reproducible and controllable size distributions. The zeta potential values were +35 and +40 mV for Cu and CuO NPs, respectively. A zeta potential of ≥30 mV in absolute value corresponds to the high stability of the nanoparticle colloids in solution [64]. The presence of copper in the sample of Cu NPs was shown by EDX spectroscopy (Figure 2b). The presence of Cu and O was also confirmed by EDX spectroscopy in a sample of CuO NPs (Figure 2d).

**Figure 3:**
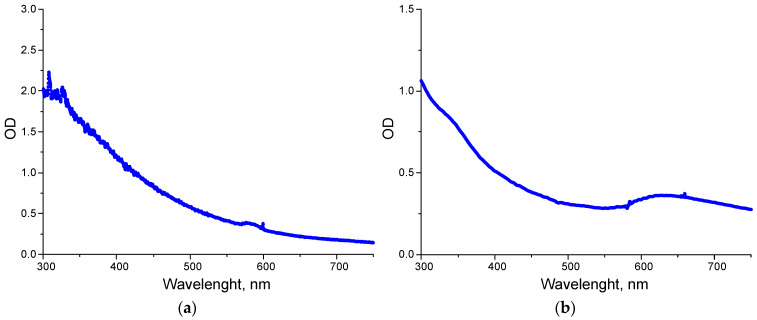
UV-vis spectra of colloids of Cu NPs in acetone (**a**) or CuO NPs in water (**b**).

The absorption spectra of Cu NPs and CuO NPs in the UV–visible region exhibited similar characteristics: increased absorption in the 300–500 nm range and absorption peaks in the 550–750 nm range (Figure 3). In the low-frequency range, the Cu absorption peak was localized within a narrower region of 560–600 nm, which is consistent with the literature data on the absorption peak of Cu NPs at 575–578 nm, which corresponds to the plasmon absorption band of non-oxidized Cu NPs [65,66]. The broadband in the UV-Vis spectrum at 600–700 nm (Figure 3b) of CuO NPs may indicate surface plasmon resonance or band edge transitions. An absorption band at 570–700 nm range is commonly observed and associated with the localized surface plasmon resonance of spherical nanoparticles [67]. This band typically corresponds to the CuO band edge transition [68]. The increased absorption of CuO in the ~300–400 nm region is consistent with the literature [69].

### 3.2. Physicochemical Properties of Materials Based on PLA and Nano-Sized Cu or CuO

The search for materials suitable for 3D printing in biomedical applications remains a pressing issue. In this study, we investigated how the physicochemical properties, antibacterial activity and cytotoxicity of PLA depend on the type of Cu or CuO nanoparticles incorporated. We prepared composites based on PLA + 0.1% Cu NPs and PLA + 0.1% CuO NPs.

The nanoparticle concentration was selected based on our previously published data as the most effective for a wide range of nanoparticle compositions and polymer matrix characteristics [55,70].

**Figure 4:**
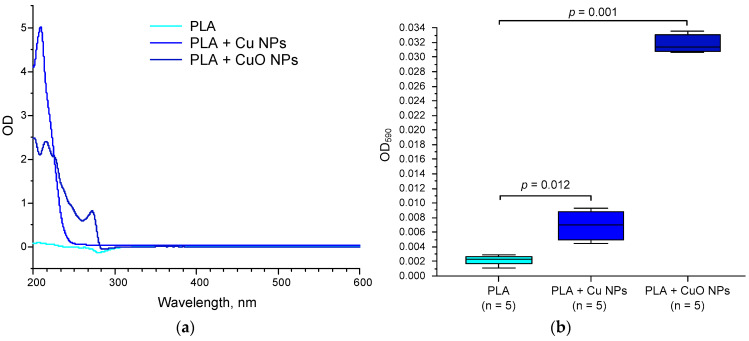
The optical properties of PLA (cyan), PLA + Cu NPs 0.1% (blue) and PLA + CuO NPs 0.1% (dark blue): UV-vis spectra in the range of 200–600 nm (**a**) and OD590 values (**b**). The data are presented as medians (vertical lines within the box), 25th and 75th percentiles (the bottom and top of the box), and maximum and minimum values (bars above and below the box). The *p*-values were calculated based on Mann–Whitney test results. Sample sizes (n) are indicated below the corresponding columns.

The physicochemical properties of the PLA-based materials containing Cu or CuO NPs are presented below. To determine the chemical composition, the UV–visible spectra of the materials were analyzed (Figure 4).

The addition of the low-frequency component increased the absorption of the polymers in the 200–250 nm range for Cu NPs and in the 200–280 nm range (Figure 4a). The absorption peak of the materials in the 200–300 nm range coincides with the absorption peaks of CuO NPs obtained via other methods [71]. However, the increase in absorption in the UV region is too significant for an addition of 1/1000 of the mass; therefore, these changes are likely caused not only by the introduction of NPs but also by alterations in the chemical structure of the polymer matrix. To further assess the penetration of NPs into the polymer matrix, the absorption of the material was measured at a wavelength of 580 nm in the absorption region of Cu NPs and CuO NPs [72]. The addition of 0.1% Cu NPs and CuO NPs increased D_580_ by a factor of 3 and 11, respectively, compared with PLA without NPs (Figure 4b), rising from 0.002 to 0.006 and 0.034, respectively. Changes in D_580_ indicate the incorporation of Cu and CuO nanoparticles into the polymer matrix.

The significant decrease in D_580_ of Cu or CuO NPs inside the PLA polymer matrix compared to D_580_ in solutions may be due to a red shift (longer wavelengths) and widening of the resonance band of the localized surface plasmon resonance peak due to the higher refractive index of PLA (*n* = 1.45–1.50) compared to the solvents, water (*n* = 1.33) or acetone (*n* = 1.36) [73,74]. A red shift in the plasmon resonance peak may also be caused by NP aggregation in the polymer matrix [75].

**Figure 5:**
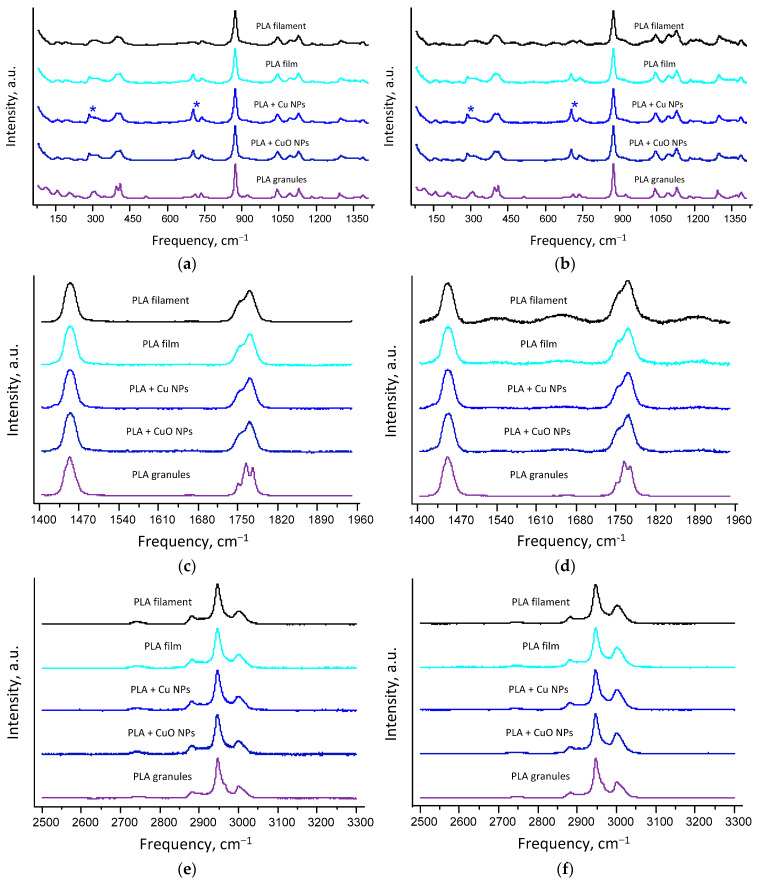
Raman scattering spectra of PLA in the form of a raw filament (black), in the form of a film without NPs (cyan), PLA films + Cu NPs (blue), PLA films + CuO NPs (dark blue), and L-PLA granules without NPs with a crystallinity of 85–87%, recorded at an excitation wavelength of 785 nm (**a**,**c**,**e**) or 532 nm (**b**,**d**,**f**) with a spectral resolution of 1.5 cm^−1^: (**a**,**b**) in the range 80–1410 cm^−1^, normalized to the peak intensity of the line at 874 cm^−1^; (**c**,**d**) in the range 1405–1950 cm^−1^, normalized to the peak intensity of the line at 1454 cm^−1^; (**e**,**f**) in the range 2500–3300 cm^−1^, normalized to the peak intensity of the line at 2947 cm^−1^. The lines of dichloromethane residues at frequencies of 287 and 703 cm^−1^ are marked in the figure with blue asterisks.

An important characteristic of polymers, including PLA, is their degree of crystallinity. To assess the degree of crystallinity, Raman spectra were recorded and analyzed for PLA, PLA + Cu NPs and PLA + Cu NPs (Figure 5). As a positive control with varying degrees of crystallinity, Raman spectra of commercially available L-PLA pellets were obtained and analyzed. The spectra of PLA without NPs exhibited the shape and peaks characteristic of L-PLA, with no additional peaks. In the Raman spectra of PLA + Cu NPs samples, additional Raman spectra lines of dichloromethane residues were observed; dichloromethane was used as a solvent during the preparation of the PLA + Cu NP samples. The most intense Raman spectra lines of DCM at frequencies of 287 and 703 cm^−1^ are marked with blue asterisks (Figure 5a,b).

Raman spectra lines from other solvents were not detected in the spectrum of the PLA + Cu NP films. No additional peaks were detected in the Raman spectra of PLA + CuO NPs, apart from the peaks of the functional groups of L-PLA. The spectra of the samples under investigation show only slight changes upon the introduction of low-concentration CuO or Cu, and this conclusion holds true for both excitation wavelengths used. The low-concentration lines are not visible due to the very low mass fraction of 0.1%

It is known that as the degree of crystallinity of L-PLA increases, the intensity of the band around 400 cm^−1^ rises, and the band splits into a doublet with peaks at 396 cm^−1^ and 411 cm^−1^ [76]. In this case, the intensity of the high-frequency component increases more rapidly than that of the low-frequency component. According to the results of quantum chemical calculations, the L-PLA vibrational line at 411 cm^−1^ corresponds to the scissoring vibrations of the O−C(H)−C(H_3_) bonds, whilst the L-PLA vibrational line at 396 cm^−1^ corresponds to the rocking vibrations of the O−C=O bonds.

**Figure 6:**
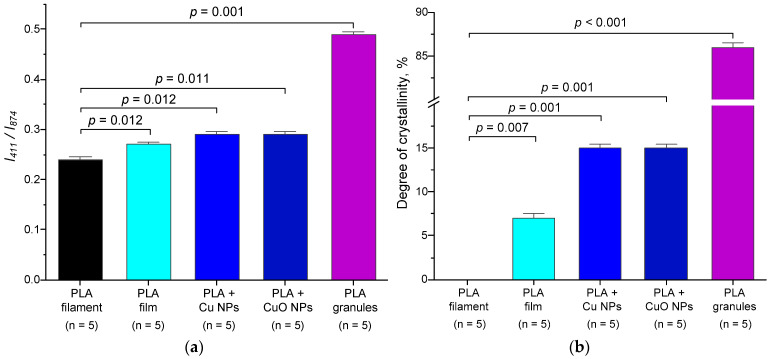
Raman spectral properties of PLA filaments (black), PLA film without NPs (cyan), PLA + Cu NPs 0.1% (blue), and PLA + CuO NPs 0.1% (dark blue), PLA granules (purple): ratios *I*_411_/*I*_874_ (**a**), degrees of crystallinity (**b**). Data are presented as mean ± SE. Demonstrated *p*-values were calculated based on Mann–Whitney test results. Sample sizes (n) are indicated below corresponding columns. *p* ≥ 0.05 not shown.

The intensity of the line at 874 cm^−1^ is a convenient reference for normalizing Raman spectra in the 80–1410 cm^−1^ range. This line has a distinct intensity and shows little overlap with other lines. According to quantum chemical calculations, it corresponds to C−O−C symmetric stretching vibrations in the molecular skeleton. It is known that the ratio *I*_411_/*I*_874_ of the peak intensities of the PLA Raman lines at frequencies of 411 and 874 cm^−1^ increases with increasing PLA crystalline content, and this relationship is linear. In the case of fully amorphous L-PLA, the value of the *I*_411_/*I*_874_ ratio is approximately 0.24, whereas in the case of L-PLA with a degree of crystallinity of 86%, the value of this ratio is approximately 0.49 (Figure 6a,b).

Spectral analysis showed that the *I*_411_/*I*_874_ ratio for the initial PLA film corresponds to a fully amorphous sample with *I*_411_/*I*_874_ = 0.24. For the PLA film without Cu NPs, *I*_411_/*I*_874_ is 0.27, which corresponds to a degree of crystallinity of 7%. Consequently, partial recrystallisation of the PLA sample occurs during dissolution and drying. For the PLA + Cu NPs and PLA + CuO NPs, an increase in *I*_411_/*I*_874_ to 0.29 and in the degree of crystallinity to 15% is observed. Consequently, the incorporation of Cu or CuO NPs into PLA doubles its degree of crystallinity compared to PLA without NPs. This result is expected, as film formation and drying took place at room temperature, whereas achieving high degrees of crystallinity requires annealing at high temperatures and a controlled film cooling process [77].

The Raman spectra of the samples under investigation are shown in the range from 1415 to 1950 cm^−1^ (Figure 5c,d). This range includes the stretching band of C=O bonds in the 1730–1800 cm^−1^ region. This range can be used to estimate the D-PLA content in a sample within the range of 1–5% *w*/*w* [77,78]. However, an accurate calculation of the D-PLA content is only possible when the degree of crystallinity is ≥50%; therefore, this method is not applicable to the film samples obtained in this study.

In the Raman spectra of the samples under investigation, in the range from 2500 to 3300 cm^−1^ (Figure 5e,f), lines at a frequency of 2947 cm^−1^ are clearly observed. This line corresponds to the symmetric stretching vibrations of the CH_3_ groups in PLA molecules, as demonstrated in [77]. The shape of the Raman PLA spectrum in this region depends only slightly on the excitation wavelength. Our results are consistent with the literature.

A comparison of the Raman spectra of PLA films containing nanoparticles, measured at several points on the sample surface, revealed no differences, indicating that the sample structure is sufficiently homogeneous. The results of the Raman spectroscopy analysis are identical for both excitation wavelengths: 532 and 785 nm.

**Figure 7:**
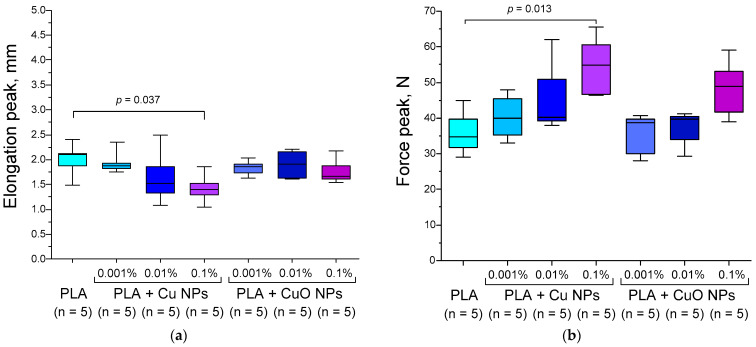

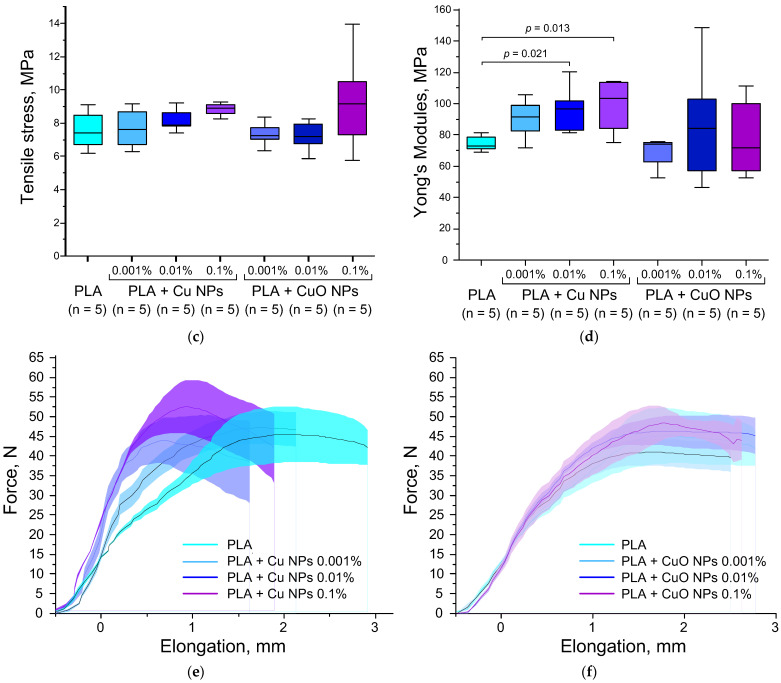
The mechanical properties of PLA without NPs (cyan), PLA + Cu NPs 0.001% (sky blue), PLA + Cu NPs 0.01% (blue), PLA + Cu NPs 0.1% (light purple), PLA + CuO NPs 0.001% (light blue), PLA + CuO NPs 0.001% (dark blue), or PLA + CuO NPs 0.1% (dark purple): average elongation peak (**a**), force peak (**b**), tensile stress (**c**), and Young’s modules (**d**), average stress–strain curves of PLA + Cu NPs (**e**) or PLA + CuO NPs 0.1% (**f**) materials. Data (**a**–**d**) are presented as medians (vertical lines within the box), 25th and 75th percentiles (the bottom and top of the box), and maximum and minimum values (bars above and below the box); data (**e**,**f**) are presented as mean ± SE. Demonstrated *p*-values were calculated based on Mann–Whitney test results. Sample sizes (n) are indicated below the corresponding columns. *p* ≥ 0.05 are not shown.

It is well known that incorporating nanoparticles into a polymer matrix and/or altering the degree of crystallinity can modify the mechanical properties of the polymer. In particular, this is described for composites based on *k*-Carrageenan/PVA/MgZnO. In this case, proportional dependence of the degree of crystallinity on the concentration of introduced NPs is shown [79]. The nature of these changes can vary. We investigated the effect of incorporating Cu or CuO nanoparticles into a PLA matrix on its mechanical properties (Figure 7). The increase in the degree of polymer crystallinity upon the addition of Cu or CuO NPs occurs mainly due to the mechanism of heterogeneous nucleation. Nanoparticles act as active centres (nuclei), facilitating the ordering of macromolecules and crystallite growth, which leads to an increase in the density, rigidity, and thermal stability of the material [80,81]. Copper nanoparticles have a high surface energy of 0.9–1.7 J/m^2^ for a size of 20 nm [82,83], so polymer chains on their surface are more easily arranged into ordered structures (lamellae) than in a pure solution or liquid form. Nanoparticles reduce the activation energy required to initiate the crystallization process, which allows a larger volume of polymer to enter the crystalline state. With the addition of Cu or CuO NPs, a greater number of nuclei are formed, which leads to the formation of a smaller but denser crystalline structure [84,85]. The change in mechanical properties, such as Young’s modulus, with the addition of Cu NPs is consistent with this mechanism. Differences in the degree of crystallinity of PLA + Cu NPs and PLA CuO NPs can be explained by differences in the composition of the NPs and their sizes. With increasing size, the surface energy usually decreases [86]. In the present work, the hydrodynamic diameter of the synthesized Cu NPs was three times smaller than that of CuO NPs (25 nm vs. 80 nm) (Figure 1). The surface energy of CuO NPs (size 20 nm) is ~0.7–0.9 J/m^2^ [87,88], which is 0.1–0.9 J/m^2^ (or ~1.3–2.4) times lower compared to Cu NPs of the same size [82,83]. The differences in the degree of crystallinity of PLA + Cu NPs (15%) and PLA + CuO NPs (7%) are twice the expected difference in surface energy. However, we understand that this observed pattern is due to the combined effects of composition and size on the surface energy of the NPs.

We found that quantitative characteristics, particularly force peak and elongation, showed increasing and decreasing trends depending on the concentration of Cu or CuO NPs. Doping Cu NPs (0.001, 0.01%) or CuO NPs (0.001, 0.01%) did not change the elongation, force peak, and maximum tensile strength of the PLA compared to a control polymer.

We found that the incorporation of 0.1% Cu NPs into PLA reduced the maximum tensile strength of the PLA by approximately 30% compared with the polymer without nanoparticles (Figure 7a). This is consistent with the literature, which indicates that the addition of Cu NPs or CuO NPs to the PLA matrix typically reduces elongation at break, increasing the material’s brittleness by restricting the mobility of the polymer chains [38,89]. However, adding 0.1% Cu NPs to PLA increased its peak force from 35–45 N to 45–60 N compared to PLA without NPs (Figure 7b), tensile stress from ~7.6 to ~9 MPa (Figure 7c), and Young’s modulus from 70–80 MPa to 90–115 MPa (Figure 7d). Adding 0.1% CuO NPs to PLA caused a less pronounced increase in all of these characteristics, which did not reach statistical significance. Young’s modulus proved to be the most sensitive to the composition type and concentration of NPs. For CuO NPs at all varied concentrations, Young’s modulus did not differ from that of the control PLA without NPs. The addition of 0.001% Cu NPs led to a tendency toward an increase in Young’s modulus, but this did not reach statistical significance (*p* = 0.06). Increasing the concentration of doped Cu NPs to 0.01% resulted in an increase in Young’s modulus compared to PLA without NPs. However, PLA + Cu NPs at 0.01% did not demonstrate a significant decrease in elongation. Our data are consistent with the dependence of the Young’s modulus of polymers, using polypropylene as an example, on the concentration of introduced Cu NPs [90,91]. Thus, by adjusting the concentration of Cu NPs introduced into the PLA matrix, its mechanical properties can be finely tuned.

**Figure 8:**
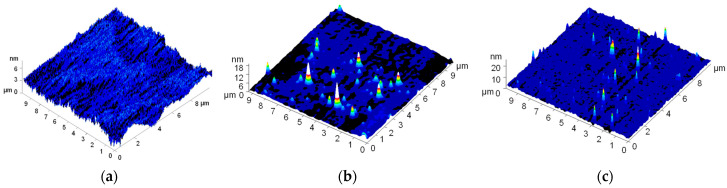
Three-dimensional reconstructions of material surfaces obtained by AFM: (**a**) PLA without NPs, (**b**) PLA + Cu NPs 0.1%, (**c**) CuO NPs 0.1%. Colours indicate the degree of surface roughness. Height scales are shown in the left-hand corner of each image.

An analysis of the surface roughness of materials following solidification and grinding, using the AFM method, is presented below (Figure 8). The control PLA, produced without the addition of NPs, exhibited a smooth surface free from cracks, tears and other defects. The *Y*-axis profile roughness of these samples did not exceed 6 nm (Figure 8a). Following the addition of 0.1% Cu NPs to the PLA polymer matrix, particles with a width of approximately 600–700 nm and a height of 18–20 nm were observed (Figure 8b). The addition of 0.1% CuO NPs to the PLA polymer matrix resulted in the formation of clusters on the surface with a diameter of 300–400 nm and a height of 18–20 nm (Figure 8c).

**Figure 9:**
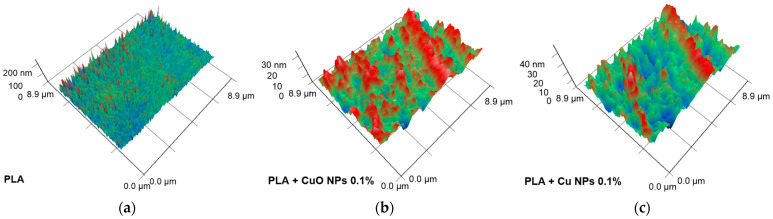
Three-dimensional reconstructions of the phase differences in transmitted light through polymer materials produced by MIM: (**a**) PLA without NPs, (**b**) PLA + Cu NPs 0.1%, (**c**) CuO NPs 0.1%. The colours indicate the degree of surface roughness of the materials.

Modulation–interference microscopy (MIM) was used to investigate the distribution of low-frequency components in the PLA polymer matrix (Figure 9). Control samples of PLA without Cu nanoparticles exhibited a random distribution of the phase difference in the transmitted light, indicating the internal homogeneity of the polymer matrix and the absence of microscopic bubbles, internal cracks and other defects.

Following the addition of 0.1% Cu nanoparticles to the polymer matrix, distinct regions with increased phase difference were observed, indicating the formation of rounded nanoparticle clusters within the polymer matrix. The cluster sizes ranged from 0.1 to 0.4 µm. Some of the clusters coalesced into larger, elongated formations with lengths exceeding 8 µm. A similar pattern was observed for the PLA + 0.1% CuO PNs samples; however, the sizes of the individual clusters were larger, ranging from 0.2 to 0.5 µm.

The low-temperature method developed has enabled the incorporation of Cu or CuO NPs into the PLA polymer matrix, as confirmed by UV–visible spectroscopy, AFM and MIM. Spectroscopic analysis revealed the presence (albeit in small quantities) of Cu or CuO within the PLA matrix (Figure 4). The AFM technique has made it possible to detect surface irregularities in polymers (Figure 8). Presumably, these were aggregates of Cu or CuO NPs. The dimensions of the nanoparticle aggregates ranged from 0.1 to 0.5 µm in width and 1 to 18 nm in height. The observed aggregates were 1–5 nanoparticles wide, but their height was significantly less than half the diameter of the Cu nanoparticles or a quarter of the diameter of the CuO nanoparticles. Consequently, the nanoparticles are largely embedded within the polymer matrix and aggregated. To investigate the distribution of Cu or CuO nanoparticles within the polymer matrix, we used MIM (Figure 9). We found that Cu and CuO nanoparticles formed aggregates within the polymer. According to the SEM results, the sizes of the nanoparticle aggregates ranged from 0.1 to 1 µm, which is confirmed by AFM data. The sizes of Cu and CuO nanoparticle aggregates, both on the PLA surface and within the polymer matrix, were comparable, indicating that the distribution of copper-containing nanoparticles within the PLA matrix is independent of their degree of oxidation.

Heterogeneity in the incorporation of NPs into the polymer matrix may be the cause of heterogeneity in the mechanical properties across different areas of the polymer surface or bulk. However, it is worth noting that the sizes of Cu or CuO NP aggregates on the PLA surface or within the polymer matrix do not exceed 0.4 µm (Figure 7 and Figure 8). The proportion of the projected polymer volume containing Cu or CuO NP aggregates does not exceed ~36%, according to MIM data. According to AFM data, the clusters occupy no more than 5% of the surface area. Furthermore, as evidenced by MIM data, the aggregates are distributed fairly uniformly throughout the polymer. Consequently, their contribution to changes in the physical and antibacterial properties should also be uniform across the entire polymer surface. The small size of the aggregates, their uniform distribution, and low total area indicate the absence of major changes within the polymer and the absence of large gradients in crystallinity change in the polymer matrix. The aggregation of nanoparticles of various natures within the polymer matrix has been widely described in the literature. However, the presence of NP aggregates in the concentration range up to 5%, as a rule, increases Young’s modulus, alters tensile stress, and changes elongation at break [37,92]. Our results on the effect of NP aggregates on the mechanical properties of PLA are consistent with the literature data.

Hydrophobicity and hydrophilicity are important properties of polymers used in biomedical applications. Significant hydrophobicity in materials can hinder tissue adhesion during prosthetic implantation, whereas hydrophilicity, conversely, helps the materials to maintain the proper morphology of the cells that come into contact with them [93].

**Figure 10:**
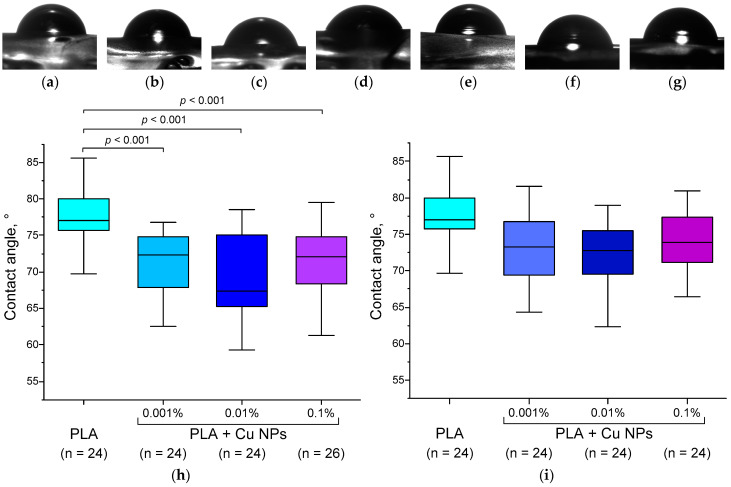
The dependence of hydrophilicity on material composition: a representative photograph of water droplets on the surface of PLA without NPs (**a**), PLA + Cu NPs 0.001% (**b**), PLA + Cu NPs 0.01% (**c**), PLA + Cu NPs 0.1% (**d**), PLA + CuO NPs 0.001% (**e**), PLA + CuO NPs 0.01% (**f**), or PLA + CuO NPs 0.1% (**g**); the average contact angle of PLA + Cu NPs (**h**): PLA (cyan), PLA + Cu NPs 0.001% (sky blue), PLA + Cu NPs 0.01% (blue), or PLA + Cu NPs 0.1% (light purple). The average contact angle of PLA + CuO NPs (**i**): PLA + CuO NPs 0.001% (light blue), PLA + CuO NPs 0.01% (dark blue), or PLA + CuO NPs 0.1% (purple). Data are presented as medians (vertical lines within the box), 25th and 75th percentiles (bottom and top of the box), and maximum and minimum values (bars above and below the box). Demonstrated *p*-values were calculated based on Mann–Whitney test results. Sample sizes (n) are indicated below the corresponding columns. *p* ≥ 0.05 not shown.

The hydrophilicity of the materials was assessed based on the contact angle (Figure 10). The average contact angle for PLA without Cu nanoparticles was 76–80°. The addition of Cu at concentrations ranging from 0.001% to 0.1% nanoparticles significantly reduced the contact angle to 68–76°, indicating an increase in the hydrophilicity of the polymer surface. The effect of Cu NPs on contact age did not depend on Cu concentration. Our results are consistent with the literature, which states that nanoparticles generally alter the contact angle by enhancing its surface energy and wettability, typically reducing the contact angle [94,95,96]. As a rule, the addition of NPs changes the contact angle of the polymer proportionally to the concentration of the introduced NPs [97]. In our case, the dependence of the contact angle on the concentration of Cu NPs is virtually absent. This is probably due to the low concentration of Cu NPs (<1%) studied in this work, compared with the NP concentrations (2–30%) described in the literature [96,97]. It is worth noting that the contact angle of PLA without NPs is less than 90°, which corresponds to a hydrophilic material. Doping Cu O NPs (0.0001–0.1%) did not change the contact age of PLA. The incorporation of CuO nanoparticles into PLA resulted in a tendency towards a decrease in the contact angle, but this did not reach statistical significance. This result appears logical, as oxides are less hydrophilic. The increased hydrophilicity of PLA + Cu NPs and PLA + CuO NPs compared to PLA indicates improved potential biocompatibility for biomedical applications.

### 3.3. The Physicochemical Properties of Water and Aqueous Solutions Following Incubation with Materials

**Figure 11:**
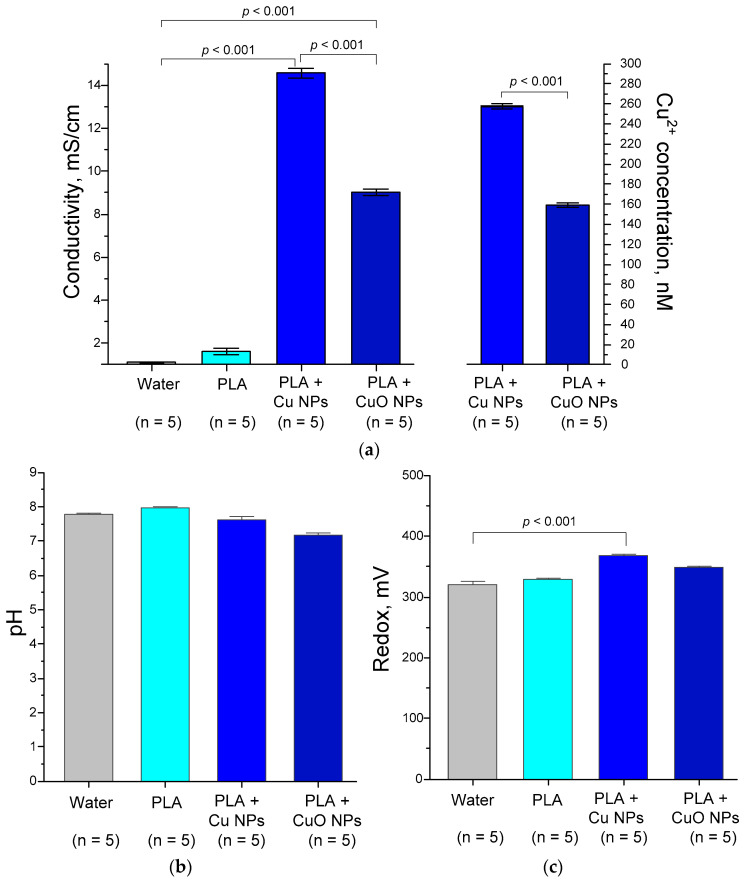
Physicochemical properties of water after 72 h of exposure to PLA (cyan), PLA + Cu NPs 0.1% (blue), PLA + CuO NPs 0.1% (dark blue), or without a material (grey): water conductivity (left) and calculated Cu2+ concentrations (right) (**a**); average pH (**b**); average redox (**c**). Data are presented as means ± SD. Demonstrated *p*-values were calculated based on two-sample *t*-test results. Sample sizes (n) are indicated below the corresponding columns. *p* ≥ 0.05 not shown.

The release of Cu^2+^ ions into an aqueous solution is proposed as a potential mechanism for the antibacterial activity of Cu/CuO nanoparticles; these ions, in turn, exert a genotoxic effect and/or inactivate bacterial enzymes. To test the hypothesis regarding the release of Cu^2+^ from the resulting polymeric materials, we conducted a series of experiments to assess the release from PLA + Cu NPs or PLA + CuO NPs by measuring changes in electrical conductivity. Prolonged exposure of the materials may affect the pH and redox potential of the solution. Changes in these parameters may be critical in the case of biomedical applications. Therefore, we assessed the effect of chronic (72 h) incubation of PLA + Cu NPs or PLA + CuO NPs on the electrical conductivity, pH and redox potential of water at a physiological temperature of 37 °C (Figure 11).

The electrical conductivity of the water without a sample (control) at the start of the experiment was 0.09–0.15 µS/cm, which corresponds to values typical of deionized water (Figure 11a). After 72 h of incubation with constant stirring at +25 °C, a slight increase in electrical conductivity to 1.5 mV was observed. This increase is insignificant and may be caused by the dissolution of carbon dioxide in the water. After 72 h of incubation with PLA + 0.1% Cu NPs, the electrical conductivity of the water increased to ~14.6 mV, corresponding to a Cu^2+^ concentration of ~257 nM. For water after incubation with PLA + 0.1% Cu NPs, the increase in electrical conductivity was less pronounced and amounted to 9 mV, corresponding to ~159 nM Cu^2+^. The non-toxic concentration of Cu^2+^ ions for humans in drinking water is generally considered to be below ~20–21 µM [98]. Antibacterial concentrations of Cu^2+^ ions in water typically range from 1 µM to 1 mM [99,100]. A number of studies indicate a lower threshold of minimum inhibitory concentrations of Cu^2+^ in water for bacteria: 50–300 µg/mL or 800–4700 nM Cu^2+^ [101,102]. Consequently, the release of Cu^2+^ from the nanoparticles in the resulting polymeric materials is possible and may even contribute to the potential antibacterial activity of the composite materials; however, other mechanisms must be involved to ensure a significant antibacterial effect. One of the main mechanisms of antibacterial activity of Cu or CuO nanoparticles is Cu^2+^-dependent ROS generation in aqueous solutions [103]. Although ROS generation by this mechanism is slow in deionized water, the rate of Fenton-like ROS generation reactions can be significantly accelerated in salt solutions, especially chlorides. Moreover, in contrast to the classical Fenton reaction, Cu^2+^-catalyzed OH radical generation occurs faster at neutral pH [104]. These conditions describe nutrient broths and/or physiological fluids. The main substrate in Cu^2+^-dependent Fenton-like reactions is H_2_O_2_. Under aerobic conditions, ROS will be generated by bacteria in culture [105]. Consequently, the involvement of Cu^2+^ in Fenton-like ROS generation in bacterial cells may be significantly greater than in deionized water. Consequently, the release of Cu^2+^ from the nanoparticles in the resulting polymeric materials is possible and may even contribute to the potential antibacterial activity of the composite materials; however, other mechanisms must be involved to ensure a significant antibacterial effect. Thus, Cu^2+^ ions may contribute to the antibacterial activity of the materials we developed, but, as described in Section 3.4, the primary antibacterial mechanisms are likely LRPS generation and secondary intracellular ROS generation.

PLA is a non-polar material with high resistance [106]. Pure PLA conducts electricity very poorly and does not release charged particles into water when incubated in it. The introduction of charge carriers is a separate and not fully resolved problem [107]. The introduction of pure PLA into water should not cause a change in its electrical conductivity, which was demonstrated in the present study. We also did not use mixed nanoparticles or additional surfactants. Traces of solvents used to transfer the NPs into PLA were also excluded, as their lines were not visible in the Raman spectra. DCM is an exception, but its contribution to the change in electrical conductivity was excluded. DCM’s relatively high permittivity (approximately 9.1 at 20 °C) allows it to dissolve organic compounds, but does not provide electrolytic dissociation. Therefore, changes in electrical conductivity can only be caused by the dissociation of Cu^2+^ cations. Polymer vials were specially used for incubation and measurements to exclude the dissociation of Na^+^, K^+^, and Ca^2+^ cations from glass.

The pH of the control water without materials after 24 h of incubation at 37 °C (measurements were taken at 25 °C) was neutral to slightly alkaline, ranging from 7.6 to 7.8 (Figure 11b). Incubation with any of the materials under investigation did not cause a change in the water’s pH. The redox potential of the control water (without materials) after 72 h was ~320–330 mV (Figure 11c). This redox potential value is typical for deionized water after the dissolution of atmospheric gases in it. Incubation with PLA + Cu NPs increased the redox potential to ~380–390 mV, which may be due to an increase in the concentration of Cu^2+^ in the water.

Among the best-characterized mechanisms underlying the antibacterial activity of nanoparticles, including those of copper and its oxide, are the generation of reactive oxygen species (ROS) and/or the redox modification of biopolymers (DNA and proteins [108,109,110]. H_2_O_2_ is one of the most stable species capable of crossing the membrane. OH radicals, on the other hand, are unable to penetrate cells through the lipid bilayer of the membrane, but possess an extremely high reactivity [111,112,113]. Moderate amounts of ROS are generated in all aerobic cells and play a role in regulating cell division, differentiation and migration [114]. Excessive production of ROS and/or disruption of antioxidant systems may lead to the development of ‘oxidative stress’, resulting in genotoxic effects, protein inactivation and lipid peroxidation [115]. These processes increase the risk of developing tumours and mutagenesis, and accelerate the body’s ageing process [114,116,117,118]. Therefore, assessing the generation of reactive oxygen species is important both for determining the mechanism of antibacterial action and for evaluating the risks of toxicity to humans. The oxidative modification of DNA and proteins may, on the one hand, confer antibacterial activity; on the other hand, it may contribute to the toxicity of these materials to humans.

**Figure 12:**
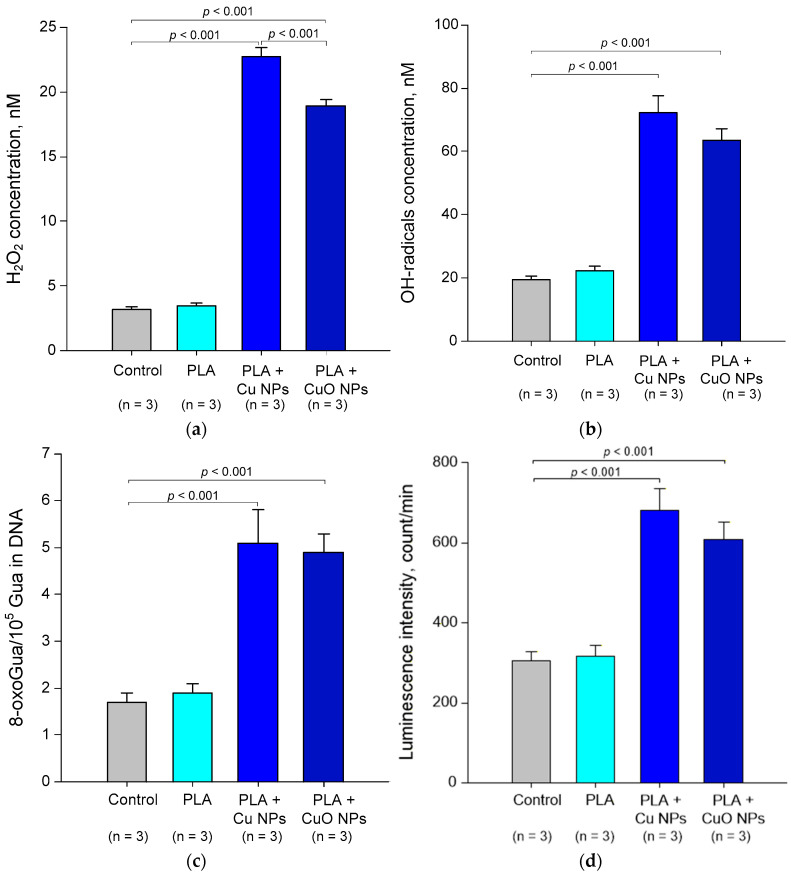
The dependence of reactive species generation rate from material composition: H_2_O_2_ concentration after 3 h at 40 °C (**a**), OH radicals after 2 h at 80 °C (**b**), 8-oxoguanine after 3 h at 80 °C (**c**), or long-lived reactive protein species after 2 h at 40 °C (**d**). Exposure of water solution to PLA (cyan), PLA + Cu NPs 0.1% (blue), PLA + CuO NPs 0.1% (dark blue), or without a material (grey). Data presented as the means ± SD. The demonstrated *p*-values were calculated based on two-sample *t*-test results. Sample sizes (n) are indicated below the corresponding columns. *p* ≥ 0.05 not shown.

We conducted two series of experiments to investigate the generation of reactive species. In the first series, we assessed how the generation rates of the reactive oxygen species H_2_O_2_ and OH radicals varied with material composition. In the second series, we assessed the rates of generation of DNA and protein oxidation products based on the concentrations of 8-oxoguanine and LRPS, respectively (Figure 12).

PLA without the addition of HCN did not result in an increase in H_2_O_2_ production compared with the control (Figure 12a). However, the concentration did not exceed 3.5 nM. PLA + 0.1% Cu NPs increased H_2_O_2_ generation to 22.7 nM (6.49 times higher than the control values). PLA + 0.1% CuO NPs had a similar effect on H_2_O_2_ generation, but to a lesser extent. The H_2_O_2_ concentration was only 18.9 nM (5.4 times higher than the control values). PLA without the addition of nanoparticles did not cause an increase in OH radical generation compared to the control (Figure 12b). The control concentration of OH radicals did not exceed 22.2 nM. PLA + 0.1% Cu NPs and PLA + 0.1% CuO NPs increased OH radical generation to 72.4 nM and 63.4 nM, respectively (3.26 and 2.86 times higher than the control values). It is worth noting that all generated concentrations of H_2_O_2_ and OH radicals did not exceed tens of nM. These ROS concentrations are ~1000 times lower than the normal level for a eukaryotic cell, and ~10^6^ orders of magnitude lower than those toxic to eukaryotes and aerobic organisms. [119]. It follows, therefore, that the materials obtained will not induce a full-scale ROS-dependent oxidative response, and their potential antimicrobial activity is attributable to other ROS-independent mechanisms or a combination thereof.

The concentration of 8-oxoguanine in the control solution did not exceed 2 per 10^5^ DNA guanines. Both materials, PLA + 0.1% Cu NPs and PLA + 0.1% CuO NPs, increased the concentration of 8-oxoguanine to 5/10^5^ DNA guanines (Figure 12c). In human lymphocytes, the normal concentration of 8-oxoguanine does not exceed 0.3/10^5^ dG [120,121]. On the one hand, in conditions associated with oxidative stress, its levels rise to 1.3/10^5^ dG [122]. In this case, the substances produced should be regarded as potentially toxic. On the other hand, the ‘normal’ concentration of 8-oxoguanine varies considerably between different cell types. In intestinal cells, the normal concentration is 1.6/10^5^ dG, comparable to that observed in the presence of 0.1% PLA + Cu NPs or PLA + CuO NPs 0.1% [123]. Significantly higher concentrations of 8-oxoguanine are detected in cancer cells 10.7/10^5^ dG [124,125]. In this case, PLA + 0.1% Cu NPs or PLA + 0.1% CuO NPs can be considered safe provided that they pass cytotoxicity tests. Furthermore, eukaryotic cells possess active 8-oxoguanine repair systems [126,127]. We therefore assume that the generation of 8-oxoguanine in the presence of the developed materials in vivo will not reach levels toxic to eukaryotic cells.

The rate of LRPS generation was assessed by measuring the intensity of spontaneous chemiluminescence in an aqueous BSA solution (Figure 12d). The spontaneous chemiluminescence of the control water was 305 counts/min. PLA without NPs did not significantly alter this characteristic. PLA + 0.1% Cu NPs and PLA + 0.1% CuO NPs increased the chemiluminescence of the water to 681 and 608 counts/min, respectively. This indicates an increase in LRPS generation in the presence of these materials. LRPS are reactive compounds and may contribute to the formation of new secondary free radicals, such as H_2_O_2_, and lead to indirect, delayed and prolonged damage to DNA, lipids and proteins [117,128,129,130,131]. It can therefore be expected that the antimicrobial activity of the developed materials is provided by the generated LRPS [117,128,129,130,131]. PLA + 0.1% Cu NPs showed a tendency towards more pronounced LRPS generation compared with PLA + 0.1% CuO NPs; however, this trend did not reach statistical significance. Summarizing the results obtained, it can be suggested that the materials PLA + Cu NPs 0.1% and PLA + CuO NPs 0.1% may induce ‘oxidative stress’ of bacteria, mainly due to the modification of biopolymers (increased generation of 8-oxoguanine and LRPS).

### 3.4. Antibacterial Activity and Cytotoxicity

**Figure 13:**
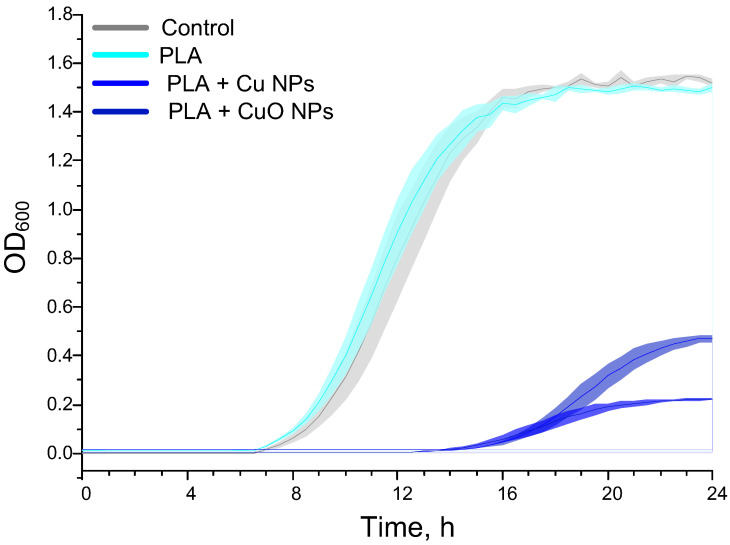
Growth curves of E. coli during cultivation in control conditions (without samples), in presence of PLA (cyan curve), PLA + Cu NPs 0.1% (blue curve), or PLA CuO NPs 0.1% (dark curve). Data presented as mean OD600 values (solid lines) ± SE (transparent areas) (n = 3 for each sample).

The results of the assessment of how bacterial growth rates depend on the composition of materials are presented below (Figure 13). Under control conditions, the *E. coli* culture exhibited a lag phase lasting 6–7 h. This was followed by linear and logarithmic growth phases lasting 2 and 6 h, respectively. To compensate for the effects of possible absorption by the added materials and to facilitate interpretation, the OD_600_ value of each culture at the zero point was subtracted from all subsequent values for that culture. The increase in optical density at the maximum was ~1.5 OD_600_ units. Incubation of the bacterial culture with PLA without NPs did not significantly alter the characteristics of the *E. coli* growth curves. Upon incubation of the bacterial culture with PLA + 0.1% CuO NPs, a significant prolongation of the lag phase and a decrease in the maximum OD_600_ of the culture were observed. The lag phase increased to 13 h (6 h longer than in the control). The maximum OD_600_ decreased to 0.4–0.5 (approximately 60% lower than in the control). When the bacterial culture was incubated with 0.1% PLA + Cu NPs, a comparable prolongation of the lag phase and an even greater reduction in the maximum OD_600_ of the culture were observed compared to the case of 0.1% PLA + CuO NPs. The maximum OD decreased to 0.18–0.20 (approximately 86–88% lower than in the control). The data obtained indicate significant bacteriostatic properties of the PLA + Cu NPs and PLA + CuO NPs; however, doping PLA with Cu NPs confers a more pronounced (48–50% higher) antibacterial activity on the material compared with CuO NPs.

**Figure 14:**
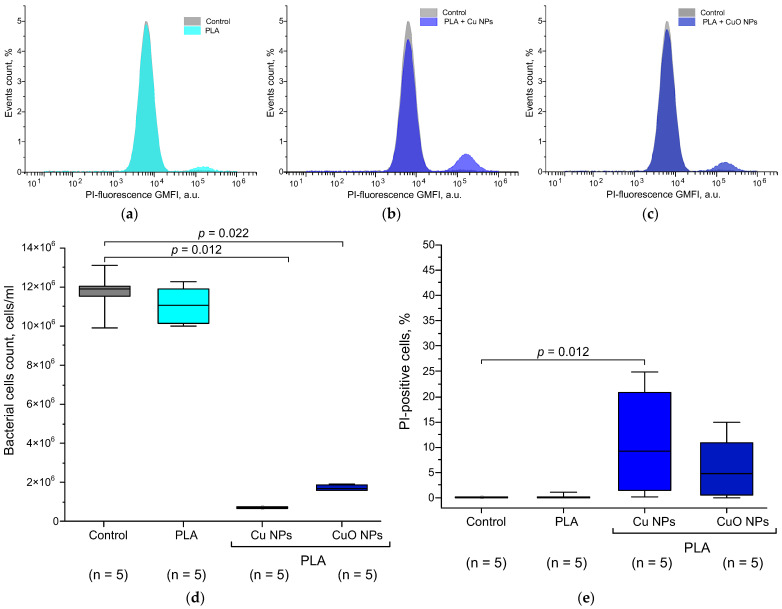
Data on the antibacterial activity of Cu and CuO NPs assessed by flow cytometry: examples of histograms of *E. coli* cell distribution by PI fluorescence intensity after 24 h of incubation in the presence of PLA without NPs (**a**), PLA + Cu NPs 0.1% (**b**), or PLA + CuO NPs 0.1% (**c**). Distribution histograms of control *E. coli* (incubation for 24 h without materials) are shown in grey. Averaged values of bacterial cells concentration, (**d**) and the proportion of dead cells (PI-positive) (**e**) after 24 h of incubation with the test materials. Data are presented as medians (vertical lines inside the box), 25th and 75% percentiles (bottom and top of the box), maximum and minimum values (bars above and below the box). The demonstrated *p*-values were calculated based on Mann–Whitney test results. Sample sizes (n) are indicated below the corresponding columns.

The results of the detailed assessment of the antibacterial effect of materials based on PLA and nano-sized Cu and CuO are presented below (Figure 14). PLA without NPs did not have an effect on either the bacterial growth rate or the number of PI-positive cells. The addition of CuO NPs had a significant bacteriostatic effect, reducing the cell count in the culture >60 times compared to the control. (Figure 14d), but did not have a statistically significant bactericidal effect (Figure 14e). However, a tendency towards an increase in the number of dead PI-positive cells after incubation with PLA + Cu NPs was observed. After incubation of the *E. coli* culture with PLA + Cu NPs 0.1%, a more pronounced bacteriostatic effect was observed compared to PLA + CuO NPs 0.1%. In the case of PLA + Cu NPs 0.1%, the decrease in the concentration of bacterial cells was ≥100 times compared to the control and ≥3 times compared to PLA + CuO NPs 0.1%. In addition to the pronounced bacteriostatic effect, PLA + Cu NPs 0.1% demonstrated a statistically significant bactericidal effect and increased the number of PI-positive cells from 1% to 10%.

Flow cytofluorometry data and spectral growth curves demonstrated similar antibacterial effects for PLA + Cu NPs 0.1% and PLA + CuO NPs 0.1%, confirming their bacteriostatic and, in the case of PLA + Cu NPs 0.1%, bactericidal activity. The numerical data on bacterial concentrations obtained by OD_600_ assessment and flow cytometer counting differ slightly. These differences are due to the specifics of the counting procedure. OD_600_ assessment involves assessing the contribution of whole bacterial cells, their fragments, extracellular matrix, spores, gas microbubbles, and other objects that can contribute to turbidity, scattering, and OD over a wide wavelength range. Flow cytometer counting is more precise, with particle size filtering during counting, eliminating the counting of cell fragments, their matrix, etc. A more accurate count of bacterial cells allowed for more accurate detection and evaluation of the bacteriostatic activity of the obtained materials.

When testing the antibacterial properties of PLA + 0.1% Cu NPs and PLA + 0.1% CuO NPs, we found a dependence of bacteriostatic and bactericidal properties on the type of nanoparticles. The addition of 0.1% Cu NPs or 0.1% CuO NPs to PLA provides the polymer with a stable bacteriostatic effect: bacterial growth was inhibited by more than 60% and 80%, respectively. According to literature data, bacteriostatic concentrations of CuO NPs range from 100 to 5000 μg/mL, which corresponds to 0.01–0.5% (*w*/*w*) [132,133,134,135].

For Cu NPs, the range of antibacterial concentrations lies in the higher range of 10–50 mg/mL (1–5% *w*/*w*) [136]. Our findings on the higher antibacterial activity of CuO NPs compared to Cu NPs are consistent with literature data. The conjugation of Cu or CuO NPs with polymers, using chitosan as an example, significantly reduces inhibitory and bactericidal concentrations by two to three orders of magnitude [137,138]. Differences between the antibacterial activity of Cu NPs and CuO NPs can be explained by differences in shape, size, or zeta potential. Metal oxide NPs with positive zeta potential values exhibit the most pronounced antibacterial properties compared to uncharged or negatively charged NPs [139]. The maxima of the zeta potential distribution for Cu NPs and CuO NPs were in the region of +31 mV and +42 mV, respectively. However, the pronounced antibacterial properties of Cu NPs compared to CuO NPs are not consistent with their zeta potential values.

**Figure 15:**
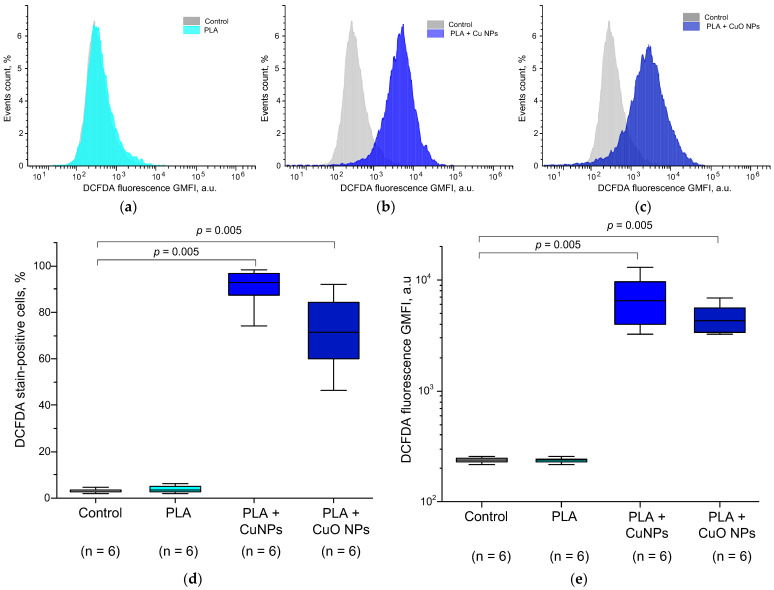
Data on the ROS production of Cu and CuO NPs assessed by flow cytometry: examples of histograms of *E. coli* cell distribution by DCFDA fluorescence intensity after 24 h of incubation in the presence of PLA without NPs (**a**), PLA + Cu NPs 0.1% (**b**), or PLA + CuO NPs 0.1% (**c**). Distribution histograms of control E. coli (incubation for 24 h without materials) are shown in grey. The averaged proportion of the bacterial cells with high DCFDA fluorescence activity (DCFDA stain-positive cells), (**d**) and the average DCFDA fluorescence intensity of total cells (**e**) after 24 h of incubation with the samples. Data are presented as medians (vertical lines inside the box), 25th and 75% percentiles (bottom and top of the box), and maximum and minimum values (bars above and below the box). The demonstrated *p*-values were calculated based on Mann–Whitney test results. Sample sizes (n) are indicated below the corresponding columns.

Since the putative mechanism of antibacterial action is the generation of ROS, we tested the possibility of this mechanism using fluorescent staining with the ROS-sensitive probe H_2_DCFDA followed by flow cytometry analysis (Figure 15).

Representative examples of histograms of the distribution of bacterial cells by DCFDA fluorescence intensity after 24 h of incubation with the test materials are shown in Figure 15a–c. Two culture characteristics were assessed. The first was the number of cells with fluorescence intensity higher than the control (Figure 15c). The second was the average fluorescence intensity across the entire culture (Figure 15d). The first characteristic shows the proportion of cells experiencing oxidative stress. The second characteristic allows us to quantify the number of ROS generated in bacterial cells. In control samples, DCFDA fluorescence intensity did not exceed 210–250 a.u., and the proportion of cells with increased DCFDA fluorescence did not exceed 2–5%. Incubation of bacteria with PLA without NPs did not change the proportion of cells with oxidative stress or the DCFDA fluorescence intensity. After incubation of *E. coli* in the presence of PLA + CuO NPs, an increase in DCFDA fluorescence intensity to ~4600 a.u. (20 times higher than in the control or PLA without NPs) was observed, and the proportion of cells in a state of oxidative stress increased to 71% (18 times higher than the control values or PLA without NPs). Incubation of E. coli with PLA + Cu NPs resulted in more pronounced oxidative stress in bacteria compared to PLA + CuO NPs. The proportion of bacterial cells in a state of oxidative stress increased to 90%, which is 23 times higher than in the control and 27% higher than after incubation with PLA + CuO NPs. The fluorescence intensity of DCFDA in the presence of PLA + Cu NPs was ~7200 a.u., which is 31 times higher than in the control and 55% higher than after incubation with PLA + CuO NPs. DCFDA has the highest sensitivity to H_2_O_2_; therefore, we assume that these ROS are generated intracellularly. Secondary generation of hydrogen peroxide with the participation of LRPS has been described in the literature [140]. Since we observed an increase in LRPS generation in the presence of both materials, we can assume that the antibacterial effect may be caused by intracellular LRPS-dependent secondary generation of H_2_O_2_.

It is likely that the differences in zeta potentials are insignificant and cannot significantly contribute to antibacterial activity. The dependence of the antibacterial properties of CuO NPs on their size is weakly expressed [29]. In special cases, Cu NPs with a size of <20 nm exhibit more pronounced antibacterial properties than NPs with a diameter of 60 nm [141]. Our data are consistent with the literature. However, smaller nanoparticles (50 nm) exhibit a short duration of antibacterial activity, while nanoparticles with a diameter of 100 nm exhibit prolonged antibacterial activity [142]. The sizes of the Cu NPs obtained in this study are in the range of 10–100 nm, which corresponds to high antibacterial activity. We believe that encapsulating Cu NPs in a PLA matrix will ensure prolonged antibacterial action due to the gradual release of copper ions and/or NPs from the polymer matrix. Thus, PLA + 0.1% Cu NPs will provide long-lasting and effective antibacterial action.

Heterogeneity in the incorporation of NPs into the polymer matrix may be the cause of heterogeneity in antibacterial properties across different areas of the polymer surface or bulk. However, for antibacterial action, Cu or CuO NP aggregation should not play a significant role. According to our data, the underlying mechanism of antibacterial action is the generation of LRPS, ROS, and Cu^2+^ release (Figure 10, Figure 11 and Figure 14). These mechanisms do not require direct contact of bacteria with the NPs, and the “uniformity” of action across different areas of the polymer surface will be ensured by diffusion in aqueous solutions.

**Figure 16:**
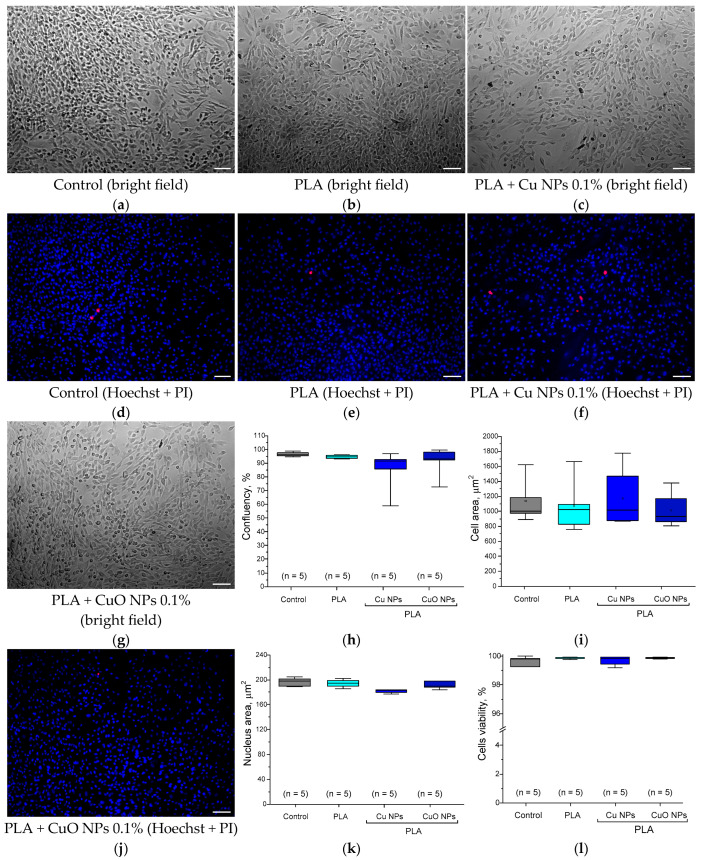
The results of the cytotoxicity assay of PLA, PLA + Cu NPs, and PLA + CuO NPs after 72 h of incubation of SHF cell culture. Examples of photomicrographs of HSF cells after 72 h of culture without materials (**a**,**d**), with PLA (**b**,**e**), PLA + Cu NPs 0.1% (**c**,**f**), or PLA + CuO NPs 0.1% (**g**,**j**) in bright field (**a**–**c**,**g**), or fluorescence channels Hoechst (blue) and PI (red) (**d**–**f**,**j**). The scale bar in the lower right corner corresponds to 50 µm. Averaged data on confluence (**h**), cell (**i**) and nuclear (**k**) areas and viability (**l**) of HSF cells. Data are presented as medians (vertical lines inside the box), 25th and 75% percentiles (bottom and top of the box), and maximum and minimum values (bars above and below the box). The demonstrated *p*-values were calculated based on Mann–Whitney test results. Sample sizes (n) are indicated below the corresponding columns.

The results of the evaluation of the potential cytotoxicity of PLA without NPs and PLA doped with Cu and CuO NPs are presented below (Figure 16). Culturing HSF cells with PLA did not cause changes in cell morphology or the appearance of a significant number of dead cells (PI-positive). The number of dead cells in the control did not exceed 1–5%, which is a normal value for eukaryotic cells. Confluence of the control cells was close to complete (95–99%). The area of individual cells was 1000–1200 µm^2^. The average nuclear area was 170–200 µm^2^. All obtained values are within the normal range for fibroblasts [143,144]. Cultivation in the presence of PLA without NPs did not cause statistically significant changes in any of the studied parameters: confluence, viability, cell and nuclear areas.

The addition of Cu or CuO NPs also had no significant effect on the parameters studied. In the presence of PLA + Cu NPs, a tendency toward a decrease in confluency and nuclear area was observed, but this did not reach statistical significance. Cell culture confluency may depend on cell concentration and adhesion ability. We expected that increasing hydrophilicity (Figure 10) for PLA + 0.1% Cu NPs would enhance cell confluency. However, we did not observe this effect. This may be due to the initial hydrophilicity of PLA after DCM treatment (contact angle < 86°) and/or a slight change in the contact angle after the addition of Cu NPs (a decrease of no more than 7–8°). Materials with a contact angle of 40–70° are considered optimal for cell adhesion [145]. However, it should be noted that the optimal wetting angle for adhesion may vary for different cell lines [145]. The cells generated in this work exhibit adequate adhesion on all the materials generated, indicating a suitable balance of hydrophilicity and hydrophobicity of PLA + Cu NP or PLA + CuO NP composites. Cell area and viability showed no significant changes. Therefore, PLA, PLA + Cu NPs, and PLA + CuO NPs do not exhibit cytotoxic activity against human cells. We chose 72 h as the classic duration for chronic nanoparticle cytotoxicity experiments [146]. Measurements at this time point allow for simultaneous assessment of acute and subchronic cytotoxicity. At this stage of the study, we assessed the fundamental feasibility of using the obtained nanomaterials with eukaryotic cells. A more detailed assessment of cytotoxicity, including long-term chronic experiments and/or 3D culturing, will be the focus of future research.

Low-temperature manufacturing methods are well suited for producing thin films, which is a convenient material format for disposable biodegradable food packaging. Furthermore, protocols for producing 3D-printed filaments based on PLA + Cu NP composites have been described in the literature [38]. PLA + Cu NP or PLA + CuO NP filaments can be used for 3D printing high-precision parts for customized biomedical applications. Filament production is thus scalable. We realize that scaling up the production of both packaging films and 3D-printed filaments is a multi-step process. However, the global market already has successful experience with the production and scale of filaments based on PLA and Cu NPs under the Copper 3D brand names “PLACTIVE AN1 Copper3D—Antibacterial” and “MDFlex Copper3D—Antibacterial” from Filament2print (Nigrán (Pontevedra), Spain). The advantage of our material is its use of a lower Cu NP concentration of 0.1% compared to commercial analogues (1–2%), which should ensure greater safety for humans and animals (especially aquatic animals).

## 4. Conclusions

In this work, a method for low-temperature incorporation of Cu or CuO NPs into a PLA polymer matrix was developed. Cu and CuO nanoparticles, introduced into the polymer matrix, were synthesized by laser ablation in solvents (water or acetone, respectively). The synthesized Cu and CuO NPs had a narrow size distribution (25 and 80 nm, respectively) and zeta potential (+31 and +42 mV, respectively). PLA + Cu NPs 0.1% and PLA CuO NPs 0.1% materials were obtained using a low-temperature embedding method in a polymer matrix by changing solvents, stirring cycles and subsequent drying. The introduction of Cu and CuO NPs at a concentration of 0.1% (*w*/*w*) did not cause the appearance of microdefects inside and on the PLA surface. Clusters of Cu and CuO NPs are clearly visualized on the PLA surface and in the bulk of the polymer matrix. Furthermore, the addition of Cu and CuO NPs increased the crystallinity of PLA 2 times compared to PLA subjected to the same manipulations without NPs. However, the change in the final crystallinity did not exceed 15%, so the addition of Cu NPs only slightly reduced the elongation of PLA + Cu NPs compared to PLA without NPs, and enhanced strength properties such as maximum tensile strength and Young’s modulus. The strength properties of the PLA + 0.1% CuO NPs material did not change relative to pure PLA. The resulting materials demonstrated a pronounced antibacterial effect, increasing the lag period (by 6 h) and reducing the maximum concentration of *E. coli* bacteria (by 80 and 60% for PLA + Cu NPs and PLA + CuO NPs, respectively) in the stationary phase compared to the control and PLA without NPs. PLA + Cu NPs demonstrated bactericidal activity, resulting in cell wall destruction in 10% of bacterial cells. Potential mechanisms of antibacterial activity, in ascending order of probability, include increased ROS generation, the release of Cu^2+^ ions, and oxidative modification of bacterial DNA and proteins. Despite their pronounced antibacterial activity, PLA + Cu NPs and PLA + CuO NPs did not alter the pH or redox potential of water and did not exhibit cytotoxic or cytostatic properties. Furthermore, Cu or CuO NPs increased the hydrophilicity of PLA, making it more suitable for biomedical applications. Based on the combined antibacterial activity and other properties, PLA + Cu NPs 0.1% is more promising for the manufacture of biodegradable products for biomedical, food, and other applications.

## Figures and Tables

**Table 1:** Key challenges in PLA + Cu or CuO NP composite development.

N°	Composition	Challenges	Disadvantages	Reference
1	PLA + Cu ion-saturated zeolite/CuO PNs 5–10%	Tensile modulus ↑ ^1^ High biocompatibility Bacteriostatic action ↑	Only 2 h antibacterial activity is shown Hydrophilicity (?) ^2^ Bactericidal activity (?) Antimicrobial action mechanism (?) Hydrophilicity (?) High concentration of 5–10% (*w*/*w*) of NPs is needed	[40]
2	PLA + CuO/Zn NPs	Antibiofilm activity Bacteriostatic activity	Bactericidal activity (?) Antimicrobial action mechanism (?) Biocompatibility (?) Mechanical properties (?)	[41]
3	PLA + Cu NPs 1–3%	Mechanical properties’ tuning ability Hydrophilicity ↑ Bacteriostatic action ↑	Biocompatibility (?) Hydrophilicity (?) Bactericidal activity (?) Antimicrobial action mechanism (?)	[42]
4	PLA + Cu NPs 1–2%	Mechanical properties tuning ability Ability to 3D printing filament manufacturing	Antimicrobial activity (?) Biocompatibility (?) Hydrophilicity (?)	[38]
5	PLA + Cu NPs 1–2% + ethanolic sumac extract	Bacteriostatic action ↑ Meet refrigerated storage period ↑	Biocompatibility (?) Hydrophilicity (?) Bactericidal activity (?) Antimicrobial action mechanism (?)	[43]
6	PLA + CuO NPs 2% + lavender essential oil	Tensile strength ↑ Bacteriostatic action ↑ Oxygen permeability ↑	Biocompatibility (?) Hydrophilicity (?) Bactericidal activity (?) Antimicrobial action mechanism (?)	[37]
7	PLA/PVA + CuO/ZIF-8 NPs	Elongation at break ↑ Bacteriostatic action ↑ Biocompatibility (by MTT test) ↑	Bactericidal activity (?) Antimicrobial action mechanism (?) Biocompatibility assay by more relevant test is needed Complex composition	[44]
8	PLA + Cu NPs or CuO NPs	Crystallization properties ↑ Bacteriostatic action ↑	Bactericidal activity (?) Antimicrobial action mechanism (?) Biocompatibility (?)	[45]
9	PLA + Cu NPs 2%	Crystallinity degree ↑ Tensile strength ↑ Elongation at break ↑ Hydrophilicity ↑ Bacteriostatic action ↑ Biocompatibility ↑	Bactericidal activity (?) Antimicrobial action mechanism (?)	[46]
10	PLA/polyhydroxybutyrate + graphene oxide/CuO NPs 3%	Bacteriostatic action ↑ Crystallinity degree tuning ability Elongation at break ↑ Young’s modulus tuning ability	Bactericidal activity (?) Antimicrobial action mechanism (?) Biocompatibility (?) Complex composition	[47]

^1^ The increasing of characteristic was shown (↑), ^2^ Data about this characteristic was not shown (?).

## Data Availability

The raw data supporting the conclusions of this article will be made available by the authors on request.

## Supplementary Materials

The following supporting information can be downloaded at: https://www.mdpi.com/article/10.3390/polym18080976/s1: Table S1: Cohen’s d vs. PLA without NPs to Figure 4; Table S2: Cohen’s d vs. PLA without NPs to Figure 6; Table S3: Cohen’s d vs. PLA without NPs to Figure 7; Table S4: Cohen’s d for contact angle vs. PLA without NPs to Figure 10h,i; Table S5: Cohen’s d vs. water without a sample to Figure 11a(left),b,c; Table S6: Cohen’s d vs. PLA with Cu NPs to Figure 11a (right); Table S7: Cohen’s d vs. control to Figure 12; Table S8: Cohen’s d vs. control to Figure 14; Table S9: Cohen’s d vs control to Figure 15; Table S10: Cohen’s d vs control to Figure 16.

## Abbreviations

The following abbreviations are used in this manuscript:

ABTS	2,2′-azino-bis-(3-ethylbenzothiazoline-6-sulfonic acid)
AFM	Atomic force microscopy
BSA	Bovine serum albumin
DCFDA	Dichlorodihydrofluorescein diacetate
DCM	Dichloromethane
DMSO	Dimethyl sulfoxide
DNA	Deoxyribonucleic acid
EDTA	Ethylenediaminetetraacetic acid
EDX	Energy-dispersive X-ray spectroscopy
ELISA	Enzyme-Linked Immunosorbent Assay
FBS	Fetal bovine serum
FS	Forward scattering
H_2_DCFDA	2′,7′-dichlorodihydrofluorescein diacetate
HSF	Human spleen fibroblasts
LRPS	Long-lived reactive protein species
NPs	Nanoparticles
PBAT	Polybutylene adipate terephthalate
PBSA	Polybutylene succinate-co-adipate
PHA	Polyhydroxyalkanoates
PI	Propidium iodide
PLA	Polylactic acid
PVA	Polyvinyl acetate
ROS	Reactive oxygen species
SS	Side scattering
TEM	Transmission electron microscopy
UV	Ultraviolet
ZIF-8	Zeolitic Imidazolate Framework-8
