# Supplementary material for: A Composite Based on L-Polylactide with Cu or CuO Nanoparticles: Physical Properties and Biological Activity
Source: Polymers (Basel). 2026 Apr 17;18(8):976. doi: 10.3390/polym18080976 (PMC13119861; doi:10.3390/polym18080976)
Supplement: Supplementary file 1 [file polymers-18-00976-s001.zip › polymers-4235954-supplementary.pdf]

Table S1 Cohen's d vs PLA without NPs to Figure 4

Parameter	PLA	PLA + Cu NPs	PLA + CuO NPs
OD_{590}	0	1,62039	1,89366

Table S2 Cohen's d vs PLA without NPs to Figure 6

Parameter	PLA filament	PLA film	PLA + Cu NPs	PLA + CuO NPs	PLA granules
I_{411}/I_{874}	0,00000	10,96438	10,76291	12,63608	55,68700
DC	0,00000	169,83129	78,86053	98,99495	121,51429

Table S3 Cohen's d vs PLA without NPs to Figure 7

Parameter	PLA	PLA + Cu 0.001% NPs	PLA + Cu 0.01% NPs	PLA + Cu 0.1% NPs	PLA + CuO 0.001% NPs	PLA + CuO 0.01% NPs	PLA + CuO 0.1% NPs
Elongation peak, mm	0.00000	-1.28772	-2.52982	-2.12280	-1.44454	-0.76642	-2.70223
Force peak, N	0.00000	1.052232828	0.643408473	0.627913581	0.790068253	1.049488744	1.86177307
Tensile stress, MPa	0.00000	-0.76483	-0.96655	1.20544	-1.57554	-1.17799	1.53482
Youg's modules, MPa	0.00000	1.16009	1.72204	1.22856	0.81004	0.84188	0.30729

Table S4 Cohen's d for contact angle vs PLA without NPs to Figure 10h.i

PLA	PLA + Cu 0.001% NPs	PLA + Cu 0.01% NPs	PLA + Cu 0.1% NPs	PLA + CuO 0.001% NPs	PLA + CuO 0.01% NPs	PLA + CuO 0.1% NPs
0.00000	-1.23404	-1.54862	-1.17570	-0.75618	-1.06586	-0.64579

Table S5 Cohen's d vs water without a sample to Figure 11a (left), b, and c

Parameter	Water	PLA	PLA + Cu NPs	PLA + CuO NPs
Conductivity, mV	0.00000	1.63299	113.44933	92.26411
pH	0.00000	4.77544	-2.03057	-13.72324
Redox, mV	0.00000	1.80939	9.91779	5.98491

Table S6 Cohen's d vs PLA with Cu NPs to Figure 11a (right)

Parameter	PLA + CuO NPs
calculated Cu^{2+} concentrations, nM	-29.68399

Table S7 Cohen's d vs control to Figure 12

Parameter	Control	PLA	PLA + Cu NPs	PLA + CuO NPs
H_2O_2 concentration. nM	0.000	0.948683298	34.20526275	34.67549919
OH-radicals concentration. nM	0.000	1.43402144	13.24986683	13.8650784
8-oxoGua/10^5 Gua in DNA	0.000	0.632455532	5.963994531	8
Luminescence	0.000	0.318166982	7.496050895	6.805123802

intensity. count/min				

Table S8 Cohen's d vs control to Figure 14

Parameter	Control	PLA	PLA + Cu NPs	PLA + CuO NPs
Bacterial cells count. cells/ml	0.000	-0.00096	-0.87791	-0.21145
PI-positive cells. %	0.00000	-0.23233	0.31713	0.39767

Table S9 Cohen's d vs control to Figure 15

Parameter	Control	PLA	PLA + Cu NPs	PLA + CuO NPs
DCFDA stain-positive cells. %	0.00000	0.56259	39.60225	7.81481
PI-positive cells. %	0.00000	0.15120	3.49443	4.63394

Table S10 Cohen's d vs control to Figure 16

Parameter	Control	PLA	PLA + Cu NPs	PLA + CuO NPs
Confluency. %	0.00000	-1.95445	-1.53169	-1.38259
Cell area. μm^2	0.00000	0.46135	1.94981	-0.00121
Nucleus area. μm^2	0.00000	-0.27868	-2.81030	-0.69947
Cells viability. %	0.00000	0.68520	0.02136	0.55338
